# Single-cell transcriptome analysis reveals subtype-specific clonal evolution and microenvironmental changes in liver metastasis of pancreatic adenocarcinoma and their clinical implications

**DOI:** 10.1186/s12943-024-02003-0

**Published:** 2024-05-03

**Authors:** Joo Kyung Park, Hyoung-oh Jeong, Hyemin Kim, Jin Ho Choi, Eun Mi Lee, Seunghoon Kim, Jinho Jang, David Whee-Young Choi, Se-Hoon Lee, Kyoung Mee Kim, Kee-Taek Jang, Kwang Hyuck Lee, Kyu Taek Lee, Min Woo Lee, Jong Kyun Lee, Semin Lee

**Affiliations:** 1 Department of Medicine, Samsung Medical Center, Sungkyunkwan University School of Medicine, Seoul, Republic of Korea; 2 Department of Health Sciences and Technology, Samsung Advanced Institute for Health Sciences & Technology (SAIHST), Sungkyunkwan University, Seoul, Republic of Korea; 3 Department of Biomedical Engineering, College of Information-Bio Convergence Engineering, Ulsan National Institute of Science and Technology (UNIST), Ulsan, Republic of Korea; 4 Department of Pathology, Samsung Medical Center, Sungkyunkwan University School of Medicine, Seoul, Republic of Korea; 5 Department of Radiology, Samsung Medical Center, Sungkyunkwan University School of Medicine, Seoul, Republic of Korea

**Keywords:** Pancreatic ductal adenocarcinoma, Liver metastasis, Single-cell RNA-sequencing, Intratumoral heterogeneity, Tumor microenvironment

## Abstract

**Background:**

Intratumoral heterogeneity (ITH) and tumor microenvironment (TME) of pancreatic ductal adenocarcinoma (PDAC) play important roles in tumor evolution and patient outcomes. However, the precise characterization of diverse cell populations and their crosstalk associated with PDAC progression and metastasis is still challenging.

**Methods:**

We performed single-cell RNA sequencing (scRNA-seq) of treatment-naïve primary PDAC samples with and without paired liver metastasis samples to understand the interplay between ITH and TME in the PDAC evolution and its clinical associations.

**Results:**

scRNA-seq analysis revealed that even a small proportion (22%) of basal-like malignant ductal cells could lead to poor chemotherapy response and patient survival and that epithelial-mesenchymal transition programs were largely subtype-specific. The clonal homogeneity significantly increased with more prevalent and pronounced copy number gains of oncogenes, such as *KRAS* and *ETV1*, and losses of tumor suppressor genes, such as *SMAD2* and *MAP2K4*, along PDAC progression and metastasis. Moreover, diverse immune cell populations, including naï﻿ve *SELL*^hi^ regulatory T cells (Tregs) and activated *TIGIT*^hi^ Tregs, contributed to shaping immunosuppressive TMEs of PDAC through cellular interactions with malignant ductal cells in PDAC evolution. Importantly, the proportion of basal-like ductal cells negatively correlated with that of immunoreactive cell populations, such as cytotoxic T cells, but positively correlated with that of immunosuppressive cell populations, such as Tregs.

**Conclusion:**

We uncover that the proportion of basal-like subtype is a key determinant for chemotherapy response and patient outcome, and that PDAC clonally evolves with subtype-specific dosage changes of cancer-associated genes by forming immunosuppressive microenvironments in its progression and metastasis.

**Supplementary Information:**

The online version contains supplementary material available at 10.1186/s12943-024-02003-0.

## Introduction

Pancreatic ductal adenocarcinoma (PDAC) is one of the most lethal diseases [1]. Most PDAC patients present with nonspecific symptoms at an advanced stage that is not amenable to curative surgery. Moreover, PDAC has clinical characteristics of early metastasis and resistance to therapy [2]. However, molecular mechanisms underlying PDAC progression, metastasis, and therapy response are still poorly understood.

With recent advances in single-cell RNA sequencing (scRNA-seq) technologies, it is now possible to characterize comprehensively the cellular composition and the transcriptomic landscape of the tumor microenvironment (TME) at the single-cell level. Therefore, scRNA-seq can be useful in studying the complex nature of intratumoral heterogeneity (ITH) and TME in PDAC for establishing novel therapeutic strategies. A few scRNA-seq studies of PDAC have been published, but the majority of these works are limited to either primary tumors [3–5] or liver metastases [6]. Furthermore, surgical specimens are usually acquired after portal vein dissection and vascular clamping, which would cause ischemic damage and autolysis of pancreatic cells. However, endoscopic ultrasound-guided fine needle biopsy (EUS-FNB) enables acquiring relatively undamaged and fresh tissue under the original physiologic status of the patient [7]. Recently, Zhang et al. reported that CEACAM5^+^/CEACAM6^+^ ductal cells are associated with poor prognosis by analyzing three cases of primary PDAC and matched liver metastases. However, these datasets offer restricted insights into PDAC evolution due to their focus on only the advanced tumor stage and a relatively small sample size [8].

Here, we performed scRNA-seq analysis of treatment-naïve 21 primary PDAC samples obtained by EUS-FNB and 7 matched liver metastasis samples acquired by percutaneous biopsy at the time of diagnosis to better understand the interplay between ITH and TME in tumor evolution and its clinical relevance in the treatment of PDAC.

## Results

### Patient characteristics

This study enrolled 21 treatment-naïve patients (Table S1). The median age was 61 (50–73 years), and 13 patients (62%) were females. Tumor clinical stages (the 8th AJCC) were 6 (29%) at stage III and 15 (71%) at stage IV. Among the 15 patients with stage IV disease, 13 had metastasis to the liver, and two had metastasis to the bone or the lymph node but not the liver. The median overall survival (OS) was 9.7 months, ranging from 0.6 to 47.8 months.

### Single-cell transcriptional landscape of primary PDACs and matched liver metastases

**Fig. 1:**
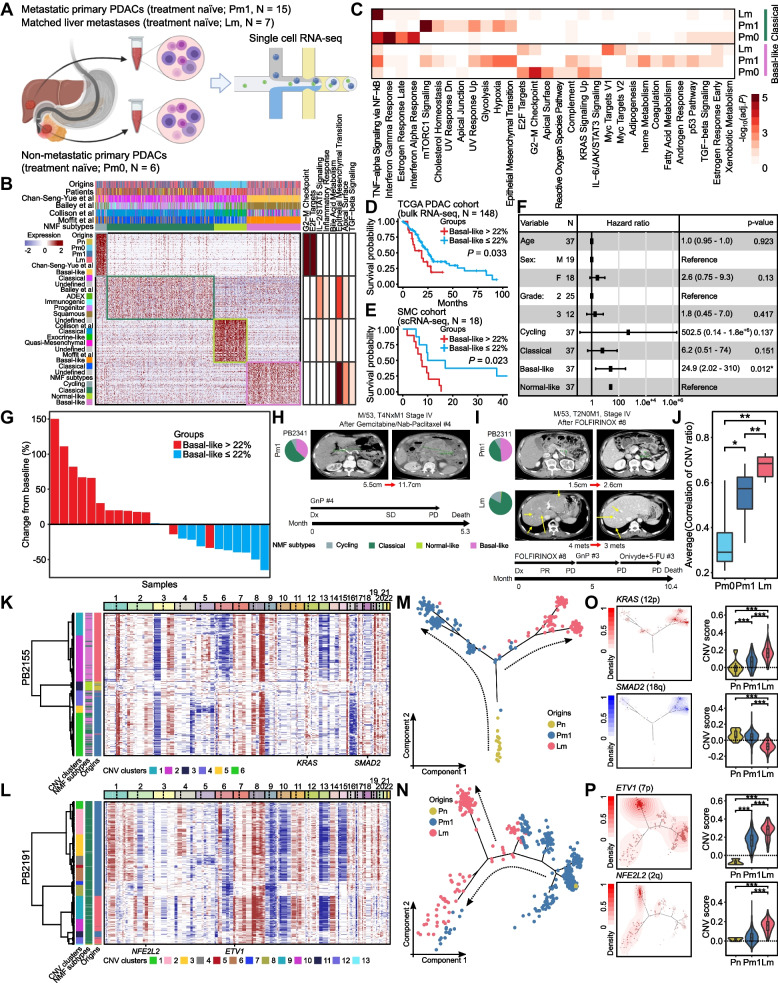
ScRNA-seq analysis of PDAC subtypes and their clinical relevance. **A** Schematic of the experimental design. ScRNA-seq was performed on PDAC samples from 21 patients, including non-metastatic PDACs (*N* = 6), metastatic PDACs (*N* = 15), and matched liver metastases (*N* = 7). **B** Heatmap showing the expression of signature genes for NMF subtypes. Each column in the heatmap corresponds to one cell and each row of the heatmap corresponds to a signature gene of four NMF subtypes. Origin, patient, NMF subtype, and previously reported PDAC classification schemes for each cell are shown at the top of the plot, and the results of GSEA for each signature gene set are shown on the right side of the plot. **C** Pathway enrichment analysis focusing on origin-specific differences within classical and basal-like subtypes. **D** and **E** Kaplan–Meier overall survival curves for PDAC patients based on the fraction of basal-like subtype in the deconvoluted TCGA PDAC RNA-seq dataset (**D**), and in their primary PDAC in our dataset (SMC cohort) (**E**). **F** Forest plot showing the estimated hazard ratios for the clinicopathologic parameters and the proportions of NMF subtypes by multivariate Cox regression analysis of combined scRNA-seq data from our cohort and the two previously published PDAC cohorts. Data are presented as hazard ratio ± 95% confidence interval. **G** Waterfall plot showing the best percentage change in the sum of the target lesions according to the RECIST v1.1. Each bar indicates a study sample, and the sample is divided into two groups: those with basal-like proportion above 22% (red) and those below (cyan). **H** and **I** The proportion of PDAC NMF subtypes and CT scan images before and after chemotherapy of PDAC patients PB2341 (**H**) and PB2311 (**I**). **J** Boxplot showing the distribution of mean CNV correlation coefficients among malignant ductal cells within origins (two-sided Wilcoxon rank sum test: *P < 0.05, **P < 0.01, ***P < 0.001). **K** and **L** Hierarchical clustering of CNV profiles in individual patients PB2155 (**K**) and PB2191 (**L**). **M** and **N** Unsupervised transcriptional trajectories of ductal cells in individual patients PB2155 (**M**) and PB2191 (**N**) colored by sample origin. Trajectory directions were indicated by arrows. **O** and **P** Dots on trajectory projections (left) were colored by copy number scores at the cellular level and overlaid with contour plots of cells with the strongest copy number variation for known cancer-associated genes in individual patients PB2155 (**O**) and PB2191 (**P**). Violin plots (right) showed copy number scores of genes by origin (two-sided Wilcoxon rank sum test: *P < 0.05, **P < 0.01, ***P < 0.001)

From these 21 patients, we obtained the following samples; 1) primary PDAC without metastasis (Pm0, *N* = 6), 2) primary PDAC with metastasis (Pm1, *N* = 15), 3) liver metastasis matched with primary PDAC (Lm, *N* = 7), and 4) adjacent normal pancreas (Pn, *N* = 5) (Fig. 1A, Table S2). We divided cells into 26 clusters and identified seven major cell types (Fig. S1A and B) (Supplementary Methods). We did not observe a batch effect, although the ductal cell clusters showed evident patient-, rather than origin-, specific gene expression profiles, which is typical in tumor cells with patient-specific copy number variations (CNVs) [5, 6] (Fig. S1C-E). When compared to previous scRNA-seq studies of PDAC, T cells were relatively enriched while fibroblasts were somewhat depleted in our data. This may be due to the way of acquiring tissues with EUS-FNB in our study, while most of the previous works used surgical resection.

### Stratification of PDAC subtypes

Sub-clustering analysis classified ductal cells into 21 subclusters, each of which was largely patient-specific (Fig. S2A). To identify distinct transcriptional programs, we applied consensus non-negative matrix factorization (cNMF) on the gene expression profiles of ductal cells. Among the four NMF subtypes determined by stability and error (Fig. S2B), three NMF subtypes were well matched with the previously reported PDAC (Supplementary Results). Therefore, we hereafter designated NMF-1 as ‘cycling’, NMF-2 as ‘classical’, NMF-3 as ‘normal-like’, and NMF-4 as ‘basal-like’.

Since the ‘epithelial–mesenchymal transition (EMT)’ pathway was involved in both classical and basal-like subtypes (Fig. 1B), we further investigated different EMT mechanisms between the two subtypes through pseudobulk-based differential gene expression analysis and gene set enrichment analysis (GSEA) among origins. Interestingly, the 'EMT' pathway was significantly activated in Pm1 of both classical and basal-like subtypes (Fig. 1C); however, actual genes involved in the 'EMT' pathway were different between the two subtypes, implying subtype-specific EMT programs might exist in PDAC (Fig. S3C). To interrogate factors potentially contributing to the subtype-specific EMT programs, we analyzed underlying transcription factors (TFs) associated with the distinct subtype-specific EMT genes in Pm1. As shown in the Fig. S3D and E, 38 and 62 TFs were identified as Pm1-specifically activated regulons in the classical and basal-like PDAC subtypes, respectively. Among the Pm1-specifically activated TFs in the classical subtype, we found that TFDP1 and CUX1 were known to respectively regulate *IGFBP2* and *MFAP5* that were detected as the classical-specific EMT-associated differentially expressed genes (DEGs) in Pm1 (Fig. S3D). We also discovered that 16 out 62 Pm1-specifically activated TFs in the basal-like subtype are involved in regulating all the basal-like-specific EMT-associated DEGs in Pm1 except *COL1A2* (Fig. S3E). Interestingly, the two Pm1-specifically activated TFs in the classical subtype and the 16 Pm1-specifically activated TFs in the basal-like subtype did not overlap with each other.

### PDAC subtypes and clinical outcomes

We identified that malignant ductal cells with different subtypes coexist in individual samples and that the proportion of each subtype varied across the samples even within the same patient (Fig. S3F). Interestingly, among seven patients having both matched primary PDAC and liver metastasis samples, five patients (PB2191, PB2264, PB2349, PB2409, and PB2410) shared common predominant subtypes, but two patients (PB2155 and PB2311) exhibited different predominant subtypes (Fig. S3F).

As the basal-like subtype of PDAC is known to be associated with a poor prognosis [9], we interrogated which proportion of basal-like subtype is associated with patients’ survival. When we tested a range of basal-like cell proportions (10% ~ 35%) while maintaining the number of samples in each group greater than 10% of total samples based on the external PDAC scRNA-seq datasets (WashU [4] and MGH [10], *N* = 25), the lowest cutoff for basal-like cell proportion showing a statistical association with survival was 22% (*P* = 0.024, Fig. S4A). In addition, we deconvoluted bulk RNA-seq data from TCGA PDAC cohort [11] (*N* = 148) using our scRNA-seq data and estimated cellular fraction of the four PDAC subtypes in each TCGA PDAC sample. When we scanned the proportions of basal-like cells and their statistical association with survival based on the deconvoluted TCGA PDAC data, 22% of basal-like cell fraction was the lowest threshold showing a statistical association with survival (*P* = 0.033, Fig. 1D and Fig. S4A). This result was also consistent in our cohort (SMC, *N* = 18) (*P* = 0.023, Fig. 1E). To estimate the prognostic relevance of NMF subtypes, we also performed multivariate Cox regression analysis for OS with age, sex, grade, and proportion of NMF subtypes in our data combined with the two previously published scRNA-seq data [4, 10]. Only the proportion of basal-like was significantly associated with shorter OS (Hazard ratio, 24.9; 95% CI, 2.02–310; *P* = 0.012; Fig. 1F).

### Evaluation of treatment response according to PDAC subtypes

We then explored the association of PDAC subtypes with treatment responses (Fig. 1G-I, Fig. S4D-G). In line with the result above, the proportion of basal-like was a key determinant for chemotherapy response. The mean best percentage change in the sum of the target lesions according to the RECIST v1.1 increased by 39% in the group contains more than 22% of basal-like subtype, and decreased by 34% in the group with less than or equal to 22% of basal-like subtype (Fig. 1G). The change in the sum of the target lesions between the two groups was statistically significant (*P* = 0.0002, Fig. S4B). In addition, the proportions of basal-like showed positive correlations with changes in tumor dimension (*r* = 0.73, *P* = 4.9 $\times$ 10^–5^) (Fig. S4C).

Figure 1H, I and Fig. S4D to G showed the 1^st^ line chemotherapeutic response according to the proportion of PDAC subtypes. For example, patient PB2341 having primary PDAC with mixed subtypes of classical (56%) and basal-like (36%) did not respond to the first four cycles of gemcitabine plus nab-paclitaxel (GnP) followed by aggressive progression, and died with an OS of 5.3 months (Fig. 1H). In addition, PB2256 with a high proportion (79%) of basal-like in primary PDAC showed rapid progression of primary mass after four cycles of FOLFIRINOX and poor response to the subsequent GnP treatment as well, with progressive disease (PD) after three cycles (Fig. S4D). Interestingly, in the case of PB2311 with different subtype compositions between primary PDAC (49% of basal-like and 42% of classical) and its liver metastasis (81% of classical), the primary tumor mass increased whereas the liver metastasis mass decreased after FOLFIRINOX treatment (Fig. 1I). On the other hand, patient PB2366 with normal-like predominant known to be excellent prognosis (93%) primary PDAC had partial response (PR) after four cycles of FOLFIRINOX treatment as the first-line chemotherapy, and the tumor was down-staged to resectable status. The patient received pylorus-preserving pancreatoduodenectomy with adjuvant FOLFIRINOX chemotherapy and has now been followed up for 44.9 months with no evidence of disease (Fig. S4E). Patient PB2032 with mixed subtypes of normal-like (58%) and classical (41%) in primary PDAC showed the best response as PR with palliative GnP treatment and was still alive with 45.6 months of OS (Fig. S4F). Furthermore, patient PB2191 with more than 90% of classical in both primary PDAC and liver metastasis had favorable responses to chemotherapeutic drugs in both sites with 17.8 months of OS (Fig. S4G).

### Dynamics of clonal evolution during PDAC progression

To understand clonal heterogeneity and evolution in the progression of primary PDAC to liver metastases, we performed CNV analysis of ductal cells from both primary PDAC and liver metastases. The CNV profiles were highly patient-specific but still largely concordant with those of TCGA PDAC cohort (Fig. S5A). We analyzed the average CNV correlation coefficients among malignant ductal cells in each sample to measure the level of clonal heterogeneity in the tumor (Fig. 1J). Interestingly, the average CNV correlation coefficients were lowest in Pm0-derived malignant ductal cells and highest in Lm-derived ones, and the differences were significant. The level of clonal heterogeneity of Pm1-derived malignant ductal cells was in between Pm0 and Lm (Fig. 1J, Fig. S5B). This result suggests that the clonal heterogeneity decreases as the tumor progresses and metastasizes to the liver.

To understand the clonal evolution of primary PDAC to liver metastasis in individual patients, we performed a hierarchical clustering analysis of ductal cells based on their CNV profiles for each patient (Fig. 1K and L, Fig. S5C and D). Overall, CNV profiles of primary PDACs and their matched liver metastases were generally concordant to each other. However, several primary PDAC- or liver metastasis-dominant CNV events were identified. We further investigated copy number changes along the tumor evolution by performing trajectory analysis of ductal cells in each patient. The lineage differentiations from Pn-derived ductal cells into Pm1- and eventually Lm-derived malignant ductal cells were well reconstructed (Fig. 1M and N, Fig. S5E and F). Notably, copy number gains of oncogenes or losses of tumor suppressor genes gradually become prevalent and pronounced along with PDAC evolution. In the cases where basal-like was predominant in liver metastases, the CNV score of *KRAS* showed a significantly positive correlation with pseudotime (Fig. 1O, PB2155: *r* = 0.26, *P* = 1.9 $\times$ 10^–6^; Fig. S5G, PB2349: *r* = 0.20, *P* = 3.0 $\times$ 10^–15^). Furthermore, malignant ductal cells with the top 10% CNV score of *KRAS* were mostly Lm-derived cells, and the CNV score of *KRAS* was also significantly higher in Lm than in Pm1 (Fig. 1O, Fig. S5G). In contrast, CNV score of tumor suppressor genes such as *SMAD2* and *MAP2K4* showed a negative correlation with pseudotime, and the copy number losses of *SMAD2* and *MAP2K4* were more prominent in the Lm of PB2155 and PB2349, respectively (Fig. 1O, Fig. S5G). In the classical dominant cases, the CNV score of *ETV1* had a significant positive correlation with pseudotime (Fig. 1P, PB2191: *r* = 0.20, *P* = 3.9 $\times$ 10^–7^; Fig. S5H, PB2264: *r* = 0.11, *P* = 4.1 $\times$ 10^–8^), but the CNV score of *KRAS* did not significantly correlate with pseudotime. The malignant ductal cells with the top 10% CNV score of *ETV1* were mostly observed in Lm, and the CNV score of *ETV1* was significantly higher in Lm than in Pm1 (Fig. 1P, Fig. S5H). The CNV scores of two other oncogenes *NFE2L2* and *PIK3CB* also showed positive correlations with pseudotime, and their CNV scores were significantly higher in Lm than in Pm1 of PB2191 and PB2264, respectively (Fig. 1P, Fig. S5H).

To further interrogate origin- and subtype-specific CNVs, single-cell CNV profiles were merged into the sample level and the frequencies of CNV events were measured across samples. Copy number gains of chromosome 2q31, 2q32, and 8q24 and losses of 6q21, 6q22, 18q12, 18q21, and 18q22 occurred more frequently in Pm1 compared to Pm0. Due to these differential copy number alterations between Pm1 and Pm0, metastasis-associated genes such as *NFE2L2* and *EXT1* were amplified while metastasis suppressor genes such as *FOXO3*, *GOPC*, *PTPRK*, *SETBP1*, and *SMAD2* were deleted in Pm1 (Fig. S6A). Interestingly, Lm showed significantly more frequent copy number gain of the chromosomal region containing *PPFIBP1* (12p11) that was known to be associated with tumor development, progression, and metastasis of PDAC than Pm1 (Fig. S6B). In addition, copy number gain of chromosome 12q11-12 containing *KRAS* occurred more frequently in the basal-like than in the classical (Fig. S6C), which was consistent with previous reports that *KRAS* amplification was more prominent in the basal-like subtype [6, 12]. However, copy number gain of chromosome 7p21 spanning *ETV1* that was reported to promote pancreatic cancer metastasis was more frequently observed in the classical than in the basal-like.

### Niche and subtype-specific characteristics of T and NK cells

We sub-clustered T/NK cells to analyze their functional characteristics in primary PDACs and liver metastases and identified a total of 24 subclusters (Fig. S7A), which were subsequently classified by the expression profiles of canonical marker genes (Fig. S7B). Differential gene expression analysis was also conducted to further characterize each subcluster (Fig. S7C and D).

**Fig. 2:**
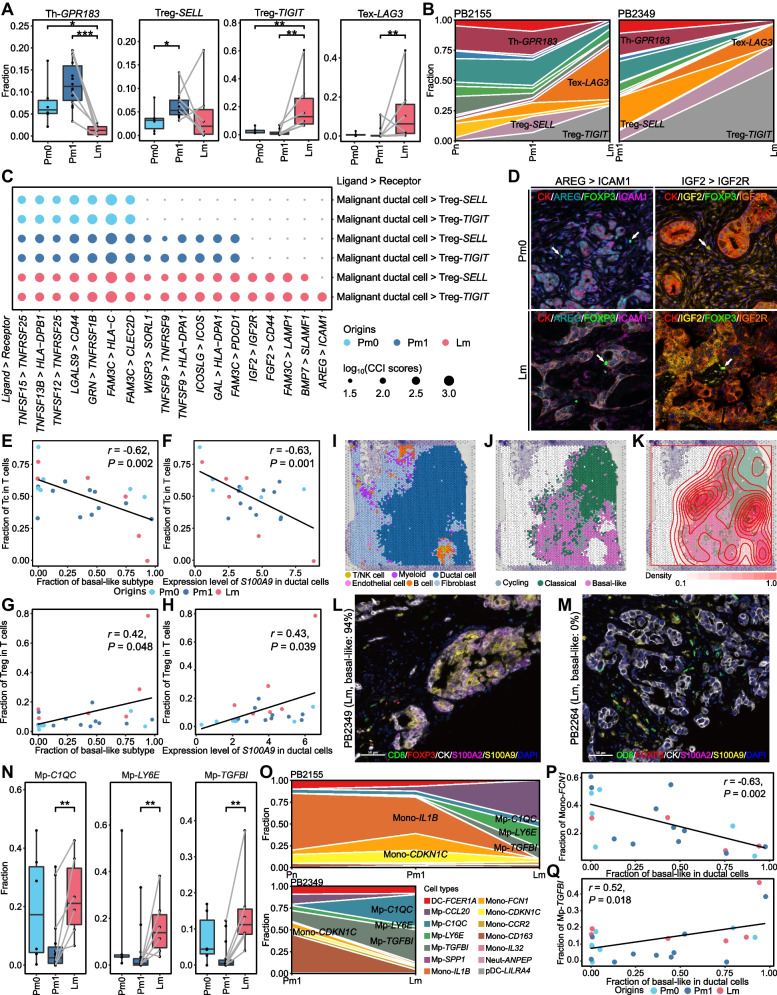
The interplay between ITH and TME in the primary PDACs and matched liver metastases. **A** Box plots indicating the percentage differences in T cell subclusters among origins (two-sided Wilcoxon rank sum test: *P < 0.05, **P < 0.01, ***P < 0.001). **B** Area plots displaying the changes in T cell subcluster composition by origin for each patient. **C** Dot plots illustrating ligand-receptor interactions between malignant ductal cells and Tregs. The size of a circle indicates an interaction score, and the color of a circle represents the origin. **D** Multiplex immunohistochemistry (IHC) showing the interaction between ICAM1 (magenta)- or IGF2R (orange)-expressing FOXP3^+^ (green) Tregs (arrows) and AREG (cyan)- or IGF2 (yellow)-expressing cytokeratin (CK)^+^ (red) tumor cells. Nuclei are counterstained with DAPI (blue). **E** and **F** Scatter plot displaying the correlation between the fraction of basal-like in ductal cells and the fraction of cytotoxic T cells in T cells (**E**), and between the expression level of *S100A9* in ductal cells and the fraction of cytotoxic T cells in T cells (**F**). **G** and **H** Pearson correlation between the proportion of basal-like among ductal cells and the proportion of Tregs among the T cell population (**G**), and between the expression level of *S100A9* in ductal cells and the fraction of Tregs among T cells (**H**). **I** and **J** Mapping of major cell types (**I**) and NMF subtypes (**J**) to spatial transcription spots from treatment naïve PDAC patient published by Zhou et al. using a robust cell type decomposition (RCTD) method. **K** The spots on the spatial transcriptome slide were colored by NMF subtypes and overlaid with contour plots of Treg enriched spots. **L** and **M** Multiplex IHC showing the expression of S100A9 (yellow) and the distribution of T cells in basal-like dominant (**L**) and classical dominant (**M**) PDAC tissues. CD8 (green) for cytotoxic T cells, FOXP3 (red) for Tregs, CK (white) for ductal cancer cells, S100A2 (magenta) for basal-like ductal cells and DAPI (blue) for nuclei were co-stained. Scale bar, 50 μm. **N** Box plots indicating the percentage differences in myeloid subclusters among origins (two-sided Wilcoxon rank sum test: *P < 0.05, **P < 0.01, ***P < 0.001). Samples from the same patients were connected by solid lines. **O** Area plots showing the change in the composition of the myeloid subclusters by origin for each patient. **P** and **Q** Scatter plot displaying the Pearson correlation between the fraction of basal-like in ductal cells and the fraction of Mono-*FCN1* (**P**) and Mp-*TGFBI* (**Q**)

When comparing Pm1 with Pm0, a naï﻿ve/resting regulatory T cell (Treg) subcluster, Treg-*SELL*, and an unstimulated natural killer (NK) cell subcluster, NK-*XCL2*, were notably enriched in Pm1. We also found that the proportions of helper T cell (Th)-*GRP183* and NK-*KLRC2* with antitumor properties were significantly reduced in Lm compared to in Pm0 and Pm1 and to in Pm1, respectively. In contrast, exhausted T cell (Tex)-*LAG3* and Treg-*TIGIT* with immunosuppressive characteristics were remarkably enriched in Lm compared to in Pm1 and to in Pm0 and Pm1, respectively. A dysfunctional NK cell subcluster, NK-*KLRC1*, was also marginally enriched in Lm (Fig. 2A, Fig. S8A). These patterns were also identified in individual patients with primary PDAC and matched liver metastases (Fig. 2B, Fig. S8B and C). The trajectory analysis of CD4^+^ T cells, CD8^+^ T cells, and NK cells confirmed that their regulatory natures and dysfunctional characteristics gradually became prominent along PDAC progression and metastasis (Figs. S9 and S10).

According to the above results, we hypothesized that the incremental activation of Tregs in PDAC evolution was potentially attributed to their interaction with malignant ductal cells, and investigated cellular interactions between ductal cells and Tregs. We discovered that Treg-activating intercellular interactions gradually established as PDAC progressed and metastasized into the liver. For example, Treg-stabilizing LGALS9-CD44 interactions between ductal cells and Tregs were identified in all three origins (Fig. 2C). However, FOXP3-inducing TNFSF9-TNFRSF9 interactions were observed in Pm1 and Lm, but not in Pm0. Interestingly, an IGF2-IGF2R interaction known to promote Treg proliferation was only observed in Lm, and another Treg-enhancing AREG-ICAM1 interaction between ductal cells and Treg-*TIGIT* was also identified uniquely from Lm (Fig. 2C). These two liver metastases-specific immunosuppressive interactions between ductal cells and Tregs were further validated using multiplex immunohistochemistry (mIHC) (Fig. 2D, Fig. S11-13).

To interrogate the association between PDAC subtypes and the immune environment, we analyzed correlations between the proportions of ductal and T cell subtypes. The proportion of basal-like was negatively correlated with the proportion of cytotoxic T cells (Fig. 2E), as previously reported by Raghavan et al. [6] and Hwang et al. [4]. Especially, *S100A9*, a basal-like signature gene known to suppress T cell proliferation by activating myeloid-derived suppressor cells, showed significant negative correlation (Pearson's correlation *r* = -0.63; *P* = 0.001) between its expression level and the proportion of cytotoxic T cells (Fig. 2F). In contrast, the proportion of Tregs was positively correlated with the proportion of basal-like (Pearson's correlation *r* = 0.42; *P* = 0.048, Fig. 2G), and the expression level of *S100A9* showed a positive relationship with the fraction of Tregs in T cells (Pearson's correlation *r* = 0.43; *P* = 0.039, Fig. 2H). To validate these results, we performed a deconvolution analysis of PDAC spatial transcriptomic data published by Zhou et al. [10] using our scRNA-data as a reference (Fig. 2I-K). Tregs were largely enriched in the regions where basal-like was predominant, and a significant positive correlation between the proportions of basal-like and Tregs in spatial transcriptomic spots was identified (Pearson’s correlation *r* = 0.135; *P* < 0.001). The distribution of CD8^+^ T cells and Tregs around basal-like ductal cells expressing S100A2 and S100A9 were further confirmed by mIHC (Fig. 2L and M, Fig. S14).

### TGFBI^hi^ macrophage shapes an immunosuppressive environment in liver metastasis

Fourteen distinct clusters of myeloid cells were identified (Fig. S15A), and each cell type were specified with previously described markers for myeloid (Fig. S15B). Pro-inflammatory monocyte (mono)-*IL1B* and anti-inflammatory mono-*CDKN1C* subclusters were significantly increased in Pm1 while Mono-*CCR2*, macrophage (Mp)-*C1QB*, Mp-*LY6E*, and Mp-*TGFBI* subclusters involved in tumorigenesis and immunosuppression were enriched in Lm compared to Pm1 (Fig. 2N and O, Fig. S15C and D).

Furthermore, the proportion of mono-*FCN1*, which is a classical monocyte involved in the initial inflammatory response, was inversely correlated with the proportion of basal-like malignant ductal cells (Fig. 2P). In contrast, the fraction of Mp-*TGFBI* with immunosuppressive properties was positively correlated with the fraction of basal-like cells (Fig. 2Q). Among 59 basal-like signature genes in ductal cells, 32 genes were negatively associated with Mono-*FCN1 and* 32 genes such as immunosuppression-related *HCAR2* and *CTHRC1* were positively correlated with Mp-*TGFBI* (Fig. S15E).

## Discussion

We have explored the landscape of diverse cellular populations in primary PDACs and their matched liver metastases to comprehensively understand the molecular mechanisms of tumor progression, metastasis, and treatment response in PDAC. Our work demonstrates the complex nature of ITH and TME of PDAC at the single-cell level and its association with chemotherapy responses and clinical outcomes. We also discovered that TME becomes more immunosuppressive and that the clonal heterogeneity of tumor decreases as PDAC progresses and metastasizes into the liver.

Several studies have investigated the characteristics of PDAC subtypes and their association with patient prognosis using bulk RNA-sequencing data [9, 12]. However, this approach has a fundamental limitation in that only dominant subtypes can be identified, making it difficult to interrogate the association between heterogeneous cellular compositions of PDAC subtypes and clinical prognosis. In this study, we revealed that even a small proportion (~ 22%) of basal-like malignant ductal cells has a detrimental effect on patients' survival and the overall response rate to chemotherapy, highlighting the advantages of scRNA-seq in estimating the exact cellular proportion of specific subtypes, which is more informative than the prevailing subtypes for predicting a patient's prognosis.

EMT has been reported to be predominantly activated in the basal-like subtype in PDAC [12]. However, when comparing primary PDACs and liver metastases separately by PDAC subtypes in our scRNA-seq data, the EMT pathway was notably enriched in both classical and basal-like ductal cells of metastatic primary PDACs, although the EMT-related genes were largely subtype-specific. Interestingly, we discovered that TFs known to regulate the subtype-specific EMT genes were also activated in a subtype-specific fashion, which implies that gene regulatory networks controlling EMT programs may be differently wired in the two PDAC subtypes, resulting in the activation of the subtype-specific EMT genes and indicating the need to develop personalized treatment strategies considering the ITH of PDAC.

Furthermore, we revealed that clonal homogeneity of tumor cells increases with PDAC progression and metastasis from the CNV analysis. Although primary PDACs and liver metastases shared most of CNVs, CNVs unique to either primary PDACs or liver metastases were also observed at a single-cell level. This result confirms that metastatic tumor cells evolve from one of clonal groups within the primary PDAC and that continuous clonal expansion occurs in both the primary and metastatic tissues even after metastasis [13]. Our analysis further identified CNV events conferring metastatic advantages. Notably, the copy numbers of cancer-associated genes such as *KRAS*, *ETV1*, and *SMAD2*, displayed a subtype-specific increase in prevalence and magnitude along PDAC progression, highlighting the importance of matched primary and metastatic samples for uncovering such patterns. In particular, our findings are in line with the assertion by Mueller et al. that *KRAS* dosage is an important contributor to PDAC progression and metastasis [14]. As previously reported, *KRAS* amplification was more prominent in the basal-like subtype than the classical subtype in our data [12]. However, in the classical subtype, we found that *ETV1* dosage exhibits a trend similar to *KRAS* dosage in the basal-like subtype, with *ETV1* gain being more widespread and pronounced along PDAC progression and metastasis. These findings provide new avenues for understanding the complex mechanisms of PDAC progression and metastasis in a subtype-specific manner.

Immunosuppressive Tregs are accumulated in PDAC and pancreatic intraepithelial neoplasia and related to metastasis and poor prognosis [3]. However, the impact of Tregs in PDAC progression and metastasis still remains elusive. We identified two Treg subclusters, Treg-*SELL* and Treg-*TIGIT,* significantly enriched in primary PDACs and liver metastases, respectively. Treg-*TIGIT* is terminally differentiated or activated Treg while Treg-*SELL* is in a naï﻿ve or resting state [15]. Notably, Treg-activating cellular interactions were established stepwise during PDAC progression and metastasis. These findings indicate that Tregs play an essential role in shaping the immunosuppressive TME of both primary PDAC and liver metastasis and that their functional characteristics become more regulatory as PDAC progresses.

CD8^+^ and CD4^+^ T cells are depleted while C1QC^+^ macrophages are predominant in the TME of the basal-like subtype-dominant cases [6]. We confirmed that the proportion of CD8^+^ T cells is negatively correlated with that of basal-like ductal cells. However, the proportions of CD4^+^ T cells and C1QC^+^ macrophages did not correlate significantly with the proportions of basal-like subtype in our scRNA-seq data. Instead, the proportions of C1QC^+^ macrophages were significantly more enriched in liver metastases than in primary PDACs. Moreover, the proportions of classical monocytes (Mono-*FCN1*) were negatively correlated with the proportions of basal-like ductal subtype. In contrast, the proportions of Tregs and immunosuppressive macrophages (Mp-*TGFBI*) were positively correlated with the basal-like ductal subtype. Therefore, these suggest that the proportions of basal-like subtype tend to be associated with more immunosuppressive environments compared to the other subtypes.

## Conclusions

Here, we provide a comprehensive catalog of ITH and TME from non-metastatic PDAC and metastatic PDAC with matched liver metastases to better understand the roles of ITH and TME in PDAC evolution at the single-cell resolution. Our study may provide an exciting entry point for developing novel therapeutic strategies taking into account the ITH and TME of PDAC.

### Supplementary Information

**Supplementary Material 1.**

## Data Availability

All single-cell RNA-sequencing raw data generated by this study have been deposited in the Sequence Read Archive (SRA, https://www.ncbi.nlm.nih.gov/sra). The data can be accessed under the accession number PRJNA948891. Processed scRNA-seq data have been deposited in National Center for Biotechnology Information (NCBI)’s Gene Expression Omnibus (GEO) and are accessible through GEO Series accession number, GSE263733. All code has been made available in GitHub at https://github.com/CompbioLabUnist/PDAC-scRNA-seq.

## Abbreviations

CNV: Copy number variation
EMT: Epithelial-Mesenchymal Transition
EUS: Endoscopic ultrasound
FNB: Fine needle biopsy
GnP: Gemcitabine plus nab-paclitaxel
GSEA: Gene set enrichment analysis
ITH: Intratumoral heterogeneity
mIHC: Multiplex immunohistochemistry
NMF: Non-negative matrix factorization
PD: Progressive disease
PDAC: Pancreatic ductal adenocarcinoma
PR: Partial response
scRNA-seq: Single-cell RNA sequencing
TME: Tumor microenvironment
tSNE: T-stochastic neighbor embedding
