# Supplementary material for: Single-cell transcriptome analysis reveals subtype-specific clonal evolution and microenvironmental changes in liver metastasis of pancreatic adenocarcinoma and their clinical implications
Source: Mol Cancer. 2024 May 3;23:87. doi: 10.1186/s12943-024-02003-0 (PMC11067162; doi:10.1186/s12943-024-02003-0)
Supplement: Supplementary file 1 — Supplementary Material 1. [file 12943_2024_2003_MOESM1_ESM.pdf]

Supplementary Information for

# Single-cell transcriptome analysis reveals subtype-specific clonal evolution and microenvironmental changes in liver metastasis of pancreatic adenocarcinoma and their clinical implications

Joo Kyung Park, Hyoung-oh Jeong, Hyemin Kim, Jin Ho Choi, Eun Mi Lee, Seunghoon Kim, Jinho Jang, David Whee-Young Choi, Se-Hoon Lee, Kyoung Mee Kim, Kee-Taek Jang, Kwang Hyuck Lee, Kyu Taek Lee, Min Woo Lee*, Jong Kyun Lee*, and Semin Lee*

*Corresponding authors.

Email: leeminwoo0@gmail.com, jongk.lee@samsung.com, seminlee@unist.ac.kr

## Supplementary Results

### Determination of PDAC subtypes

According to the gene set enrichment analysis (GSEA) of NMF signature genes, NMF-1 showed significant activation of cell cycle-related pathways, such as 'G2-M Checkpoint' and 'E2F Targets' (Fig. 1B). NMF-1 characteristics were very similar to the 'cycling' cell state reported by Krieger et al [1]. We observed significant upregulation of 'cycling' state-associated genes such as *CDK1*, *MKI67*, and *PCNA* (Fig. S2C, Table S3). Interestingly, the proportion of NMF-1 gradually increased as PDAC progressed and metastasized (Fig. S3A).

Ductal cells classified into NMF-2 were mostly identified as 'classical' subtypes (Fig. 1B). For further validation, we calculated signature scores based on previously reported PDAC subtypes for our NMF-based programs. The scores for both 'classical signature 1' defined by Chan-Seng-Yue et al [2] and 'classical' defined by Collison et al [3] were high in NMF-2 (Fig. S2D). Furthermore, we observed the highest overlap of signature genes between NMF-2 and the 'classical' subtype defined by Moffit et al [4] (Fig. S3B). GSEA analysis showed that 'IL−2/STAT5 Signaling', 'Inflammatory Response', and 'Epithelial-Mesenchymal Transition (EMT)' pathways were significantly elevated in NMF-2 (Fig. 1B).

Most of the ductal cells defined as 'exocrine-like' based on Collison et al [3] were classified into NMF-3 (Fig. 1B). The signature genes of NMF-3 considerably overlapped with those of 'endocrine/exocrine pancreas signature 5' defined by Chan-Seng-Yue et al [2] and 'ADEX' subtype defined by Bailey et al [5] (Fig. S3B). Moreover, the signature scores of three exocrine subtypes classified by Chan-Seng-Yue et al [2], Bailey et al [5] and Collison et al [3] were highest in NMF-3 (Fig. S2E). 'Bile Acid Metabolism' pathways were significantly elevated

in NMF-3 (Fig. 1B), as previously reported by Collison et al [3]. Also, NMF-3 proportions were significantly higher in Pm0 than in Pm1 and Lm (Fig. S3A).

Lastly, NMF-4 was primarily matched with the previously reported 'basal-like' subtype (Fig. 1B). The signature scores for 'basal-like' subtypes defined by Moffitt et al [4] and the 'basal-like signature 2' suggested by Chan-Seng-Yue et al [2] were both highest in NMF-4 (Fig. S2F). NMF-4 signature genes also best coincided with 'basal-like signature 2' genes defined by Chan-Seng-Yue et al [2] and 'basal-like' signature genes defined by Moffit et al [4] (Fig. S3B). Furthermore, NMF-4 signature genes were enriched with 'EMT', 'Apical Surface', and 'TGF-beta Signaling' pathways (Fig. 1B).

## Supplementary Methods

### Study patients

Patients clinically suspected to have PDAC were admitted for workup, and pathologically confirmed cases were prospectively enrolled in the Samsung Medical Center (SMC) Pancreatic Cancer Cohort. Primary PDAC specimens and metastatic liver specimens were obtained from 21 treatment naïve patients with PDAC. All patients provided written informed consent, and all samples were collected according to IRB regulations and approval (IRB No. 2014-04-061). In addition, we collected clinical data using electronic medical records regarding the patients age, sex, body mass index (BMI), Eastern Cooperative Oncology Group (ECOG) performance status, comorbidity (cardiovascular, chronic kidney disease, liver cirrhosis, other cancer, or diabetes mellitus), serum CEA level and CA 19-9 level at the diagnosis of PDAC. Tumor stage (according to the 8th edition AJCC guidelines), tumor size,

metastatic site, treatment course, and survival data were also reviewed. Ethical approval was obtained from the institutional review board of Samsung Medical Center (IRB No. 2014-04-061, 2016-05-011). Provenance and peer review Not commissioned, externally peer reviewed.

**Sample acquisition from primary PDAC using EUS-FNB**

All EUS-FNB procedures were performed under conscious sedation by the same experienced endosonographer (JKP) with fanning techniques sampling multiple areas within a lesion with each pass. GF-UE160-AL linear EUS apparatus (Olympus, Tokyo, Japan) equipped with an Aloka ProSound SSD 5000 processor (Wallingford, CT, USA) was used in all cases. EUS-FNB was performed using a linear array echoendoscope (Olympus, Tokyo, Japan) and a 22 G Acquire® FNB needle (Boston Scientific Co.). The slow-pull technique was used to remove the stylet and was followed by the application of 5 mL negative pressure for 5–10 seconds, during which at least 10 additional actuations were performed. A transgastric method was utilized for lesions in the pancreas body or tail, and a transduodenal approach was used for lesions in the head or uncinate process. For tissue retrieval, a stylet was introduced into the needle, or a 5-10 ml air-filled syringe was inserted into the needle. Normal tissue was obtained adjacent normal pancreas before approaching to tumor tissue. The extruded material was placed onto glass slides for primary gross inspection. EUS-FNB specimens were smeared on slides and fixed with 96% ethanol for cytological analysis. Other samples were placed in 10% formalin solution for histological analysis.

## Percutaneous biopsy for hepatic metastasis

Liver biopsy was performed at the same day or the next day of EUS-FNB for patients with metastatic PDAC. All liver biopsy procedures were performed percutaneously by a board-certified abdominal radiologist (MWL). Before the procedure, the radiologist carefully reviewed the CT or MR images, and the patients were positioned appropriately for the procedure based on the tumor location. In general, if the target tumor was in the liver dome, and thus the sonographic window was affected by the lung or rib shadow, the semi-erect position using a tilting table was preferred to enhance the sonographic window [6]. If not, a supine position was used for the procedure. The target lesion was localized using fusion imaging (Volume Navigation, Logiq E9, or Logiq E10; GE Healthcare, Waukesha, Wisconsin) of the real-time US and pre-acquired CT/MR images [7]. Local anesthesia was performed along the expected needle path between the skin and hepatic capsule using 2% lidocaine hydrochloride (Huons, Hwaseong, Korea). Percutaneous biopsy was performed using an 18-gauge automated biopsy needle (Acecut; TSK Laboratory, Tochigi, Japan) with the free-hand technique. A co-axial guide needle was routinely used to obtain sufficient tissue cores by repeated biopsy.

## Isolation of single cell from the specimens

All remaining tissues are collected in MACS® Tissue Storage Solution (Miltenyi Biotec., Auburn, CA, USA) on ice, and processed to the single cell dissociation immediately. Specimens were maintained in MACS® Tissue Storage Solution (Miltenyi Biotec.) during delivery on ice, and immediately processed for single cell isolation. Biopsy tissues are placed on a petri dish with a small volume of enzyme mixture solution (Tumor Dissociation Kit,

Miltenyi Biotec.), and minced into small pieces under 1-2 mm with surgical blades. Minced pieces are transferred to gentleMACS$^{TM}$ C-tube containing enzyme mixture solution. By choosing an appropriate program of gentleMACS$^{TM}$ Dissociator, tissue pieces are further dissociated three times with two interval incubations for 30 minutes at 37°C under continuous rotation using the MACSmix$^{TM}$ Tube Rotator. Resuspended samples are applied to a cell strainer (70 μm) and washed with 10 ml of DMEM/F12 media with 1% FBS. After a centrifugation at 400xg for 5 minutes, cells were resuspended in an appropriate volume of media and counted with trypan blue staining or acridine orange/ propidium iodide staining. Optionally, erythrocytes are removed by red blood cell lysis solution for 10 min before counting.

**Single-cell RNA sequencing**

We initiated the cell preparation process by employing the LUNA-FL™ Automated Fluorescence Cell Counter (logos biosystems). Detailed guidelines for cell preparation were sourced from the 10x Genomics Single Cell Protocols Cell Preparation Guide and the Guidelines for Optimal Sample Preparation flowchart. Libraries were meticulously constructed using the Chromium controller in accordance with the 10x Chromium Next GEM Single Cell 3' v3.1 protocol (10x Genomics). In brief, cell suspensions were diluted in nuclease-free water, aiming to achieve a targeted cell count of 10,000. The diluted cell suspension was mixed with a master mix and subsequently loaded onto a Chromium Next GEM chip, along with Single Cell 3' V3.1 gel beads and partitioning oil. Within the droplets formed in the chip, RNA transcripts from individual cells underwent unique barcoding and reverse transcription. Pooled cDNA molecules then underwent sequential processes, including end repair, addition of a

single 'A' base, and adapter ligation. Subsequently, the resulting products were subjected to purification and enrichment through polymerase chain reaction (PCR), ultimately yielding the final cDNA library. To ensure library quality and quantify the purified libraries, we adhered to the qPCR Quantification Protocol Guide (KAPA). Furthermore, library qualification was performed using the Agilent Technologies 4200 TapeStation from Agilent Technologies. The sequenced libraries were processed using the HiSeqX platform from Illumina to obtain the requisite data for our study.

**Single-cell RNA sequencing data processing and analysis**

Gene-Barcode matrices are produced using CellRanger v.3.0.2 (10x Genomics) pipeline with default parameters. Reads were aligned to the GRCh38 reference genome using the STAR algorithm [8]. To ensure the quality of the single-cell RNA-sequencing data, we implemented a filtering step at the individual sample level to remove low-quality cells. This filtering step was based on the number of expressed genes (average number of genes = 1,063) and the proportion of mitochondrial genes per cell (< 20%). Seurat [9] package was used for downstream analyses. The gene expression matrix for each cell was normalized using *NormalizeData* functions. The cell-cycle scores for each cell were calculated using the *CellCycleScoring* function, and expression of genes were scaled using the *ScaleData* function, which regresses on the total of UMI, fraction of mitochondria, and cell-cycle scores. The top 2,000 variable genes were selected through the *FindVariableFeatures* function, and dimensionality reduction was performed through the *RunPCA* function. For dimensionality reduction and clustering analysis, we used the *FindNeighbours* and *FindClusters* functions, and the optimal dimensionality was determined by *JackStraw* and *ElbowPlot* functions. We

used the *RunTSNE* function to project the cells into a two-dimensional space. To estimate the cell type of each cluster, we used the singleR [10] package and verified the expression levels of well-known cell type markers (ductal cells: *KRT19*, *AMBP*, *EPCAM*; fibroblasts: *COL1A1*, *COL1A2*, *COL3A1*; acinar cells: *PRSS1*, *CTRB1*, *CTRB2*; endothelial cells: *PLVAP*, *CLDN5*, *VWF*; T cells: *CD3D*, *IL7R*, *TCF7*; myeloid cells: *LYZ*, *CD14*, *C1QA*; B cell: *CD79A*, *MS4A1*, *CD79B;* naive T (Tn) cells: *SELL* and *IL7R*; helper T (Th) cells: *CD4* and *IL6R*; regulatory T cells (Tregs): *FOXP3* and *TNFRSF4*; cytotoxic T (Tc) cells: *CD8A* and *GZMK*; exhausted T (Tex) cells: *CTLA4* and *LAG3*; natural killer (NK) cells: *NCR3* and *NCAM1*). For sub-clustering by major cell type, we used the method described above and additionally removed potential doublets [11]. Differentially expressed genes were identified using the *FindMarkers* function on a normalized gene expression matrix. The raw gene expression matrix was used as a reference to deconvolve the spatial transcriptome data published by Zhou et al. [12] The deconvolution analysis was performed using RCTD [13] with doublet_mode set to "full".

**Correction for ambient RNA contamination**

During the process of isolating cells from a tissue sample, some cells may lyse because the conditions for dissociation are specific to each cell type [14]. We used SoupX [14] with default parameters to estimate and correct for cell-free mRNA contamination. Cell-free mRNA contamination was estimated and corrected in each sample.

**Curated doublet removal**

A scrublet [15] is used to infer doublets that may occur in the scRNA-seq process. The scrublet was processed independently for each sample as redundancy is determined through simulation. All parameters in scrublet were set to default and doublets were determined by the thresholds suggested by scrublet.

**Identification of PDAC subtypes by cNMF**

We defined specific transcript modules of the ductal cell using cNMF [16]. Through Seurat's *NormalizeData* function, cNMF received the normalized UMI count matrix of ductal cells as input, and the number of components (*k*) was set to a value between 2 and 15. We determined the optimal k based on the silhouette score and the Frobenius error, which measures the stability of the component.

**Copy number variation estimation**

To infer CNV at the single-cell level, we used copy number karyotyping of aneuploid tumors (CopyKAT [17]) was used. All parameters of CopyKat were set to default, and ductal cells with less than 3,000 genes were removed to identify clearer CNVs. CNV of ductal cells was estimated using myeloid cells as normal cells. To identify genomic regions that were significantly amplified or deleted between groups, we used the proportion of cells (> 70%) that exceeded a specific CNV threshold (absolute CNV score > 0.05) at the sample level. We also identified a significant difference in the frequency of CNV events between groups using the Fisher’s exact test.

**Modular score calculation**

Seurat's *AddModuleScore* function is used to calculate the gene expression module score for each cell. To determine the characteristics of NMF subtypes, we used the signature genes of the previously reported subtypes [2-5]. Regulatory, cytotoxic, and dysfunctional scores for T cells were determined using the gene set defined in Li et al. [18].

**Gene set enrichment analysis**

To investigate the differential EMT mechanisms between the basal-like and classical subtypes, we performed pseudo-bulk differential expression analysis using the raw UMI count sum of each sample. DESeq2 was used to identify differentially expressed genes by comparing each origin with other origins of the same subtype. Gene set enrichment analysis was performed using the "MSigDB_Hallmark_2020" database for genes that passed the criteria of fold change > 1.5 and p-value < 0.05 [19].

**Constructing single cell trajectories**

We estimate single cell trajectories using Monocle2 [20]. The Gene-Barcode matrix was normalized using the *EstimateSizeFactors* function and the variance of each gene was estimated using the *EstimateDispersions* function. We use DEGs identified through Seurat's

*FindAllMarkers* to sort cells. Afterwards, the dimension was reduced using *DDRTree*, and cells were sorted according to pseudotime through the *orderCells* function.

**Cell-cell communication analysis based on ligand-receptor pairs**

CellPhoneDB is an analytical tool that can infer potential receptor-ligand interactions based on the expression of receptors of one cell type and ligands of another cell type [21]. To identify the most relevant interactions between cell types, CellPhoneDB performs an interaction analysis by randomly substituting each cell type. Our results are analyzed by applying basic parameters.

**Survival analysis**

We conducted a Kaplan–Meier survival analysis to investigate the effect of the proportion of ductal cell subtype on patient survival using the survival and the survminer R packages. To validate our results, we also exploited the previously published scRNA-seq data from treatment naïve PDAC patient from Hwang et al. [22] and Zhou et al. [12], and bulk RNA-seq data of TCGA PDAC [23] patients. The proportion of NMF subtypes for each patient with TCGA PDAC was estimated using MuSiC [24]. MuSiC characterizes cellular type composition in bulk RNA-seq data from complex tissues by assigning weights to genes that exhibit inter-individual and inter-cellular consistency in scRNA-seq data. The UMI count of ductal cells defined in the SMC cohort was used to estimate the subtype proportions of TCGA PDAC samples. In addition, DEGs between subtypes were detected using Seurat's FindAllMarkers

function, and deconvolution was performed with genes with p_val_adj < 0.05 and avg_logFC > 0.25. Survival analysis was performed between the groups with high and low scores based on the estimated basal-like proportions of each sample, with the threshold increasing from 0.1 to 0.5 by 0.01. However, to reduce the effect of the imbalanced sample size in the grouping process, survival analysis was not performed if the number of samples in each group was less than 10% of the total samples. Multivariate Cox regression analysis was performed on the three PDAC scRNA-seq datasets (SMC, WashU, and MGH cohorts) using *coxph* function in survival R package. The covariates considered in this analysis encompassed age, gender (male and female), tumor stage (stage 2 and 3), and the proportion of NMF subtypes.

**Transcription factor-target gene regulatory network analysis**

Regulon analysis was investigated using SCENIC [25], an R package that analyzes the co-expression of transcription factors and their putative target genes. We assigned regulon activity scores using SCENIC's default parameters from the raw UMI count matrix. These scores were then used to construct co-expression networks. Regulon activity for each group was calculated as the average regulon activity score across all cells in the group.

**Multiplex immunohistochemical assay (mIHC)**

Further analysis of interaction between Treg and ductal cells, mIHC was done with core biopsy of liver metastases (Lm), however, Pm0 tissue from EUS FNB were not sufficient enough for mIHC and surgically resected Pm0 tissues were used. Formalin-fixed, paraffin-

embedded (FFPE) tissue sections were immunostained using the Opal Polaris 7 Color IHC Detection Kit (Akoya Biosciences, Marlborough, MA, USA), which uses individual tyramide signal amplification (TSA)-conjugated fluorophores to detect various targets, on the BOND RX autostainer (Leica Biosystems) and the following anti-human antibodies: panCytokeratin (DAKO, #M3515), FOXP3 (Abcam, # ab20034), CD8 (Ventana, #790-4460), LGALS9 (ThermoFisher, #CF806323), CD44 (Abcam, #ab157107), IGF2 (ThermoFisher, #MA5-17096), IGF2R (Abcam, #ab124767), AREG (Abcam, #ab234750), ICAM1 (ThermoFisher, #MA5407), S100A2 (Abcam, #ab109494), and S100A9 (Abcam, #ab63818). Each antibody was tested individually for its optimal position in the sequence of multiplex staining to minimize interference with previous antibody-TSA complexes or by alteration of epitopes.

Specifically, 4-μm-thick sections were deparaffinized on the Leica BOND-RX automated immunostainer (Leica Microsystems, Milton Keynes, UK) by baking for 30 min at 60 °C, soaking in BOND dewax Solution at 72 °C and then rehydrating in ethanol. Antigen retrieval was performed in heated citrate buffer (pH 6.0) and/or Tris-EDTA buffer (pH 9) for 30 min, sections were fixed with 7.5% neutralized formaldehyde (SAV Liquid Production GmbH). Each section was subjected to 5-6 successive rounds of antibody staining, each consisting of protein blocking with 20% normal goat serum (Dako) in PBS, incubation with primary Abs, biotinylated anti-mouse/rabbit secondary antibodies and Streptavidin-HRP (Dako, 50003), followed by TSA visualization with fluorophores Opal 520, Opal 540, Opal 570, Opal 620, Opal 650, and Opal 690 (PerkinElmer) diluted in 1X Plus Amplification Diluent (PerkinElmer), antibody-TSA complex-stripping in heated citrate buffer (pH 6.0) and/or Tris-EDTA buffer (pH 9) for 30 min and fixation with 7.5% neutralized formaldehyde. Thereafter, nuclei were counterstained with DAPI (PerkinElmer), and sections were mounted with PermaFluor fluorescence mounting medium (Thermo Fisher Scientific). Autofluorescence (negative

control) slides were also included, using primary and secondary antibodies and omitting the fluor tyramides. Whole slides were scanned using the Vectra-Polaris 3.0.3, a multispectral imaging system (Akoya Biosciences), at a low magnification of 10×. Quantification analysis and image capture were performed with InForm 2.6.0. and Phenochart 1.0.9 image viewer software (Akoya Biosciences).

**Supplementary Tables**

**Table S1. Baseline characteristics of study patients.**

| Characteristics | N = 21 |
|---|---|
| Age, median (range) | 61 (50-73) |
| Sex, n (%) | |
| Male | 8 (38%) |
| Female | 13 (62%) |
| BMI (kg/m$^2$), median (range) | 21.8 (16.0-29.1) |
| Performance status (ECOG), n (%) | |
| 0: fully active | 0 (0) |
| 1: light house work | 19 (90%) |
| 2: ambulatory | 1 (5%) |
| 3: limited self-care | 1 (5%) |
| DM (at the time of diagnosis) | |
| Yes | 6 (29%) |
| No | 15 (71%) |
| Pancreas mass (mm), median (range) | 37 (20.0-80.0) |
| Location (proximal), n (%) | |
| Uncinate/Head/Neck | 13 (62%) |
| Body | 2 (10%) |
| Tail | 6 (28%) |
| Stage (AJCC 8$^{th}$), n (%) | |
| III | 6 (29%) |
| IV | 15 (71%) |
| Metastasis, n (%) | |
| No metastasis | 6 (29%) |
| Liver metastasis | 13 (62%) |
| Other site metastasis, not including liver | 2 (9%) |
| Treatment modality | |
| Best supportive care only | 3 (14%) |
| Gemcitabine-based | 8 (38%) |
| FOLFIRINOX* | 10 (48%) |
| CA 19-9 (IU/mL), median (range) | 309.2 (3.11-115,048) |
| CEA (ng/mL), median (range) | 4.8 (1.09-185.48) |
| Overall survival (months), median (range) | 9.7 (0.6-47.8) |

* FOLFIRINOX: fluorouracil, leucovorin, irinotecan, oxaliplatin chemotherapy

**Table S2. Sample information.**

| Sample No. | Patient No. | Grade | Type of Sample | Type of Sample | Location of Sample | Liver Mets (Y, N) | Mets other than Liver | Number of cells | Mean UMI* | Mean genes |
|---|---|---|---|---|---|---|---|---|---|---|
| 1 | PB2032 | 2 | Primary PDAC | Pm0 | Pancreas | N | | 1989 | 11273 | 1549 |
| 2 | PB2151 | | Adjacent Normal Pancreas | Pn | Pancreas | Y | | 2773 | 6230 | 1619 |
| 3 | PB2151 | 2 | Primary PDAC | Pm1 | Pancreas | Y | | 6220 | 7327 | 2025 |
| 4 | PB2155 | | Adjacent Normal Pancreas | Pn | Pancreas | Y | | 293 | 18887 | 2279 |
| 5 | PB2155 | 3 | Primary PDAC | Pm1 | Pancreas | Y | | 1973 | 12346 | 2298 |
| 6 | PB2155 | | PDAC Liver Mets | Lm | Liver | Y | | 460 | 31211 | 4327 |
| 7 | PB2191 | | Adjacent Normal Pancreas | Pn | Pancreas | Y | | 2567 | 6634 | 1990 |
| 8 | PB2191 | 2 | Primary PDAC | Pm1 | Pancreas | Y | | 1979 | 14131 | 2858 |
| 9 | PB2191 | | PDAC Liver Mets | Lm | Liver | Y | | 373 | 40770 | 4774 |
| 10 | PB2203 | | Adjacent Normal Pancreas | Pn | Pancreas | Y | | 372 | 5652 | 1478 |
| 11 | PB2203 | 2 | Primary PDAC | Pm1 | Pancreas | Y | | 651 | 9025 | 2244 |
| 12 | PB2218 | 2 | Primary PDAC | Pm1 | Pancreas | N | Y (Bone) | 688 | 15542 | 2557 |
| 13 | PB2219 | 2 | Primary PDAC | Pm1 | Pancreas | Y | | 1772 | 10224 | 2521 |
| 14 | PB2256 | 2 | Primary PDAC | Pm0 | Pancreas | N | | 2423 | 14512 | 2932 |
| 15 | PB2264 | 2 | Primary PDAC | Pm1 | Pancreas | Y | | 1121 | 23032 | 3432 |
| 16 | PB2264 | | PDAC Liver Mets | Lm | Liver | Y | | 3066 | 24041 | 4510 |
| 17 | PB2265 | 2 | Primary PDAC | Pm1 | Pancreas | Y | | 1627 | 26826 | 4867 |
| 18 | PB2266 | 2 | Primary PDAC | Pm0 | Pancreas | N | | 2244 | 11164 | 1969 |
| 19 | PB2268 | 2 | Primary PDAC | Pm0 | Pancreas | N | | 1702 | 14058 | 2798 |
| 20 | PB2281 | 2 | Primary PDAC | Pm1 | Pancreas | Y | | 2833 | 8126 | 2056 |
| 21 | PB2286 | 2 | Primary PDAC | Pm1 | Pancreas | Y | | 2344 | 15857 | 3049 |
| 22 | PB2287 | | Adjacent Normal Pancreas | Pn | Pancreas | N | | 644 | 12930 | 1951 |
| 23 | PB2287 | 3 | Primary PDAC | Pm0 | Pancreas | N | | 2451 | 13063 | 2772 |
| 24 | PB2311 | 2 | Primary PDAC | Pm1 | Pancreas | Y | | 253 | 64445 | 6186 |
| 25 | PB2311 | | PDAC Liver Mets | Lm | Liver | Y | | 2596 | 17732 | 3357 |
| 26 | PB2341 | 3 | Primary PDAC | Pm1 | Pancreas | N | Y (M1 LN) | 3895 | 12391 | 2198 |
| 27 | PB2349 | 3 | Primary PDAC | Pm1 | Pancreas | Y | | 2713 | 9596 | 2424 |
| 28 | PB2349 | | PDAC Liver Mets | Lm | Liver | Y | | 1973 | 20460 | 4244 |
| 29 | PB2366 | 2 | Primary PDAC | Pm0 | Pancreas | N | | 2857 | 16426 | 3313 |
| 30 | PB2409 | 2 | Primary PDAC | Pm1 | Pancreas | Y | Y (Lung, Pleura) | 1953 | 11229 | 2465 |
| 31 | PB2409 | | PDAC Liver Mets | Lm | Liver | Y | | 5380 | 11059 | 2418 |
| 32 | PB2410 | 2 | Primary PDAC | Pm1 | Pancreas | Y | | 2460 | 10657 | 1800 |
| 33 | PB2410 | | PDAC Liver Mets | Lm | Liver | Y | | 2559 | 21248 | 3245 |

* UMI: unique molecular identifier

**Table S3. Signature genes of the four NMF subtypes.**

| NMF-1 | NMF-2 | NMF-3 | NMF-4 |
|---|---|---|---|
| MKI67 | FTL | CLU | CTSD |
| TOP2A | KRT19 | SERPING1 | PMEPA1 |
| TPX2 | KRT18 | SLC4A4 | TACSTD2 |
| HMGB2 | TM4SF1 | CYB5A | KRT17 |
| CENPF | CSTB | FXYD2 | PHLDA3 |
| NUSAP1 | TIMP1 | CFTR | SLPI |
| CDK1 | TSPAN8 | SNRPN | FXYD5 |
| PRC1 | AGR2 | CLDN10 | CCND1 |
| BIRC5 | CD55 | SPP1 | IER5 |
| MAD2L1 | S100A14 | SERPINA6 | S100A2 |
| UBE2C | TPM1 | GC | SOX4 |
| SMC4 | KRT7 | CXCL2 | CTSH |
| ASPM | SPINK1 | SERPINA5 | SNCG |
| KIAA0101 | S100P | PIGR | S100A9 |
| H2AFZ | ERO1A | AMBP | GJB2 |
| KIF20B | LGALS4 | EGR1 | GLTP |
| ZWINT | CLTB | GATM | AIG1 |
| CEP55 | LYZ | CES1 | CRABP2 |
| KIF23 | ANXA1 | RPS10 | PVRL1 |
| UBE2T | IER3 | METTL7A | CDKN2A |
| CDKN3 | LAMB3 | CD74 | PTGS2 |
| CCNB1 | HSPA1A | ZFP36 | NME4 |
| TK1 | CTSE | UGT2B15 | GLUL |
| ANLN | ASPH | FCGBP | ARHGDIB |
| CCNB2 | S100A16 | SLC3A1 | SLC9A3R2 |
| KIF11 | CEACAM6 | LGALS2 | IGF2 |
| HMMR | MIF | ID2 | FBLN1 |
| LMNB1 | MRPS21 | STXBP6 | FAM83A |
| PTTG1 | PDZK1IP1 | HOMER2 | MGST1 |
| NDC80 | SERPINB1 | KIF12 | GATA3 |
| CENPE | FAM3C | FGGY | KRT13 |
| CCNA2 | KRTCAP2 | NFKBIA | SCPEP1 |
| RRM2 | IL32 | RBP1 | LYPD3 |
| GTSE1 | S100A4 | CTGF | TFPT |
| CENPW | KLF2 | ASRGL1 | FSCN1 |
| NUF2 | ANXA3 | HHEX | LY6D |
| SPC25 | PLA2G16 | NDRG2 | PLAT |
| DLGAP5 | KLK11 | BTG2 | SULF2 |
| CDC20 | GCNT3 | RHOB | PLAU |
| SGOL2 | KLF4 | UGT2A3 | METRN |
| RAD51AP1 | PLAC8 | TCEA3 | GALNT1 |
| SGOL1 | HMGA1 | MUC5B | PHLDA1 |
| TYMS | VEGFA | SCGB3A1 | BCAM |
| NCAPG | TESC | CITED4 | ITM2C |
| ARHGAP11A | CLDN18 | NR4A1 | TYMP |
| TACC3 | ISG20 | ERICH5 | PTGES |
| KIFC1 | APOL1 | NR2F2 | IRX3 |
| STMN1 | HSPA1B | CLDN3 | KRT5 |
| AURKB | TFF2 | CA2 | TMEM40 |
| CDCA3 | TRNP1 | SERPINA1 | BAG3 |
| CDCA8 | TFPI | SERPINA4 | GJB6 |
| CENPK | AGR3 | C6 | CLEC2B |
| NEK2 | TIMP2 | ZC3H12A | SLC2A1 |
| PLK1 | SLC40A1 | GLIS3 | TGM2 |
| KIF2C | RPL36A | IRF1 | NAPRT |
| FOXM1 | RPL22L1 | MAFF | SEMA3C |

| | | | |
|---|---|---|---|
| CASC5 | AC090498.1 | RP1-60O19.1 | CFH |
| KIF4A | SNX9 | DNAJB1 | FLNA |
| AURKA | PON2 | SOD2 | LRRC8A |
| BUB1 | SRD5A3 | FAM3D | CAPS |
| DTYMK | SFTA2 | KCNJ15 | SYNGR1 |
| ATAD2 | RND3 | CRISP3 | GPR153 |
| TUBA1B | AREG | HLA-DPB1 | UPK3B |
| HJURP | CTSS | CYS1 | PVRL4 |
| CENPA | WFDC2 | PDGFD | AQP3 |
| KIF14 | RAB40B | BEX5 | LINC01503 |
| CKAP2L | CTSC | ALDH1A1 | LAMC2 |
| DEPDC1 | TFF1 | HABP2 | RHOD |
| PBK | CCDC68 | CFI | CDC42EP3 |
| TTK | CLDN23 | KDM6B | IGFBP3 |
| CDCA5 | TMEM176B | AGT | C16orf74 |
| ECT2 | RNASET2 | KIAA1324 | IGFBP7 |
| CKAP2 | SLC16A3 | C3 | TSC22D3 |
| MELK | LINC01133 | ZG16B | ADIRF |
| PRR11 | KCNQ1OT1 | ARSE | CETN2 |
| BUB1B | CYP2S1 | HLA-DRB1 | NMB |
| CKS1B | NDRG1 | C12orf75 | GRHL1 |
| DIAPH3 | TNFRSF12A | MT1F | TMEM45A |
| SHCBP1 | BIRC3 | HES1 | CST6 |
| ORC6 | TNFRSF21 | AQP1 | SEMA4B |
| RACGAP1 | ITGA2 | LCN2 | LAMB1 |
| CKS2 | MACC1 | TM4SF4 | SCCPDH |
| MYBL2 | GABRP | EPB41L4A | CD109 |
| FANCI | HS3ST1 | COL18A1 | ADAM8 |
| KIAA1524 | SEMA3B | BEX4 | KRT23 |
| SPAG5 | EMP1 | SORBS2 | AMIGO2 |
| SMC2 | PLAUR | ONECUT2 | HOMER3 |
| NCAPH | HMGCS1 | PLD1 | B4GALT1 |
| KPNA2 | C6orf48 | SEC11C | MBOAT2 |
| BRCA1 | RAB27B | SCTR | TGFB1 |
| CIT | ANXA10 | GPX2 | TRIM29 |
| SKA3 | MUC13 | GAMT | APOBEC3G |
| ASF1B | VNN1 | CXCL3 | FAM129B |
| TROAP | TM4SF5 | CD81 | PPL |
| MND1 | TXNRD1 | FBP1 | PRSS22 |
| CDCA2 | TPM2 | CYR61 | DUSP5 |
| CENPN | MALL | FHIT | NCCRP1 |
| NUDT1 | FAM46A | TFF3 | CHST11 |
| KIF18B | KLK10 | TACC1 | ARL4D |
| FAM83D | VSIG2 | SLC25A25 | MUC4 |
| ARHGAP11B | PNP | SFRP5 | LTBP2 |
| IQGAP3 | IL2RG | CHST9 | SERPINB5 |
| KIF15 | GSTM3 | ACE2 | CLIC3 |
| KIF18A | MMP7 | DEFB1 | ISG15 |
| ANP32E | TMEM176A | ONECUT1 | LRMP |
| KNSTRN | LMO4 | RASD1 | SNAI2 |
| UBE2S | PRSS8 | SLC12A2 | SYT8 |
| NCAPD2 | SMIM6 | CNN3 | ITGB6 |
| KIF20A | BMP2 | PPP1R1B | ZNF750 |
| NCAPD3 | ALDH2 | CXCL1 | MAP3K8 |
| BRCA2 | RAMP1 | GGT1 | SSPN |
| HELLS | AKR7A3 | FGFR3 | COL6A1 |
| HMGB3 | C4orf48 | HLA-DQB1 | MMP14 |
| RRM1 | AHNAK2 | TNFSF13B | MXRA8 |
| PHF19 | CMBL | SELENBP1 | LPCAT2 |
| SAPCD2 | TUBA4A | ID4 | MT2A |

| | | | |
|---|---|---|---|
| PKMYT1 | FAM101A | SOD3 | QPRT |
| DSCC1 | SLC3A2 | CLDN2 | DSE |
| HIST1H1B | ABHD11-AS1 | APCDD1 | MDM2 |
| CENPU | IDI1 | AKR1C3 | MUC16 |
| RNASEH2A | P4HA1 | ANPEP | DUSP23 |
| ESCO2 | FDFT1 | TCN1 | LSP1 |
| CLSPN | RP11-1143G9.4 | SNHG18 | NUAK1 |
| FEN1 | ONECUT3 | GADD45B | KRT15 |
| POLQ | EPS8L1 | FAM150B | KRT4 |
| DHFR | BST2 | MUC6 | S100A8 |
| CCDC34 | SLC25A37 | MT1E | CSTA |
| DNMT1 | PITX1 | KCNJ16 | PCDH7 |
| CENPM | XRCC4 | GYPC | IGFBP4 |
| PCNA | C10orf10 | CTNND2 | HMGA2 |
| DUT | RP11-350J20.12 | VTN | GPC1 |
| H2AFX | EGLN3 | SLC38A11 | MDFI |
| CCNF | SYT13 | NPDC1 | SRGAP3 |
| ATAD5 | EFNA1 | FOLR1 | VIM |
| MCM4 | HSBP1L1 | BCO2 | GPR87 |
| E2F8 | CDHR2 | DMBT1 | MMP2 |
| TUBB | EIF4E | NR5A2 | CTHRC1 |
| POC1A | C15orf48 | PAH | PGM2L1 |
| HIST1H4C | APOA1BP | SLC28A3 | CMTM3 |
| PSRC1 | AP1S1 | F5 | SGK1 |
| CDC6 | IRF7 | GAS6 | DCBLD2 |
| FAM111B | VIL1 | PIK3AP1 | RHOV |
| GPSM2 | MLF1 | NUAK2 | EPHA4 |
| MZT1 | SLC6A8 | ALDH1A2 | OAS1 |
| CHEK1 | PLA2G10 | TRPV6 | LINC00152 |
| RFC3 | CHPT1 | HLA-DRB5 | KRT6A |
| GGH | ITGB8 | NOSTRIN | PALLD |
| PIF1 | LAMA3 | LINC00261 | LGALS1 |
| MCM3 | HIST1H1C | NR2F1 | PDLIM7 |
| DBF4 | PRR15L | RP11-528G1.2 | LCP1 |
| STRA13 | ADGRF1 | WDR72 | RP11-54H7.4 |
| CENPH | PTPRR | PROX1 | F3 |
| CDC45 | MSLN | IFI6 | TGFBI |
| UHRF1 | CKB | FAM3B | MTSS1 |
| RAD51 | RP11-462G2.1 | FLRT2 | COL1A1 |
| RECQL4 | MUC3A | GP2 | FN1 |
| TUBA1C | HLA-DRA | MATN2 | CLDN1 |
| HIST1H1D | ODC1 | DCDC2 | FAM84A |
| GINS2 | SPNS2 | BEX1 | TNS4 |
| FAM111A | CRIP1 | FILIP1L | RHOBTB3 |
| TCF19 | TNNT1 | MAN1A1 | PYGL |
| CDT1 | C9orf3 | HLA-DMA | BCL11A |
| FAM64A | ANG | WNK2 | TTC9 |
| MCM10 | ARL14 | ACSM3 | PFN2 |
| FBXO5 | SULT1C2 | PDE5A | LRIF1 |
| RPL39L | TNFSF10 | SLC17A4 | CXCL17 |
| RFC4 | APOL2 | HLA-DPA1 | PODXL |
| MXD3 | EIF4EBP1 | VWA1 | SUSD4 |
| C21orf58 | SF3B4 | SLC5A1 | CTSL |
| BORA | HSD17B2 | EDN1 | PLOD1 |
| MCM7 | RDH10 | RARRES2 | MT1X |
| XRCC2 | DDIT4 | LYPD6B | ALDH1L1 |
| LIG1 | TMEM173 | ZNF667-AS1 | FRMD6 |
| ATP1B3 | CDC42EP5 | AKAP7 | SLC39A2 |
| BRI3BP | HK2 | NEB | NRP2 |
| MNS1 | MXD1 | MPP1 | SRGN |

| | | | |
|---|---|---|---|
| DTL | RPL17 | ANXA13 | KRT16 |
| CBX5 | RP11-841O20.2 | BAAT | ANXA8 |
| CDC25B | MMP1 | CYB5D1 | LAMA4 |
| E2F1 | ERRFI1 | RIC3 | SERPINE1 |
| CHAF1A | GPSM3 | TTR | TRIM16 |
| NMU | RNASE1 | TUSC3 | IFITM10 |
| WDR76 | AOC1 | NEURL3 | MSN |
| MCM2 | NCEH1 | PDLIM3 | ELF5 |
| MCM6 | PRSS21 | CFB | SLC1A3 |
| CDCA7 | SLCO4A1 | CDH6 | SLITRK6 |
| FABP5 | MSMO1 | HSPB8 | TMEM132A |
| ARHGEF39 | CEACAM5 | CRIP2 | LAPTM5 |
| GMNN | DDIT3 | DACH1 | HCAR2 |
| PTMS | DKK1 | PTGR1 | HES4 |
| FDPS | CES2 | PKHD1 | EPHB2 |
| HIST1H1E | ARHGAP29 | SCGB2A1 | COL7A1 |
| MCM5 | TRIM54 | NTRK2 | TP63 |
| TMEM97 | RARRES3 | ADORA1 | DSG3 |
| HIST2H2AC | RHOF | SCD5 | G0S2 |
| LDHB | CA9 | DPM3 | CDH3 |
| TNFAIP8L1 | PPP1R14D | RP11-469H8.6 | PLTP |
| CDKN2C | UPP1 | PRKACB | GPNMB |
| SLC43A3 | SAMD9 | HGD | CRLF1 |
| CCDC88A | CA12 | COLCA1 | OVOL1 |

## Supplementary Figures

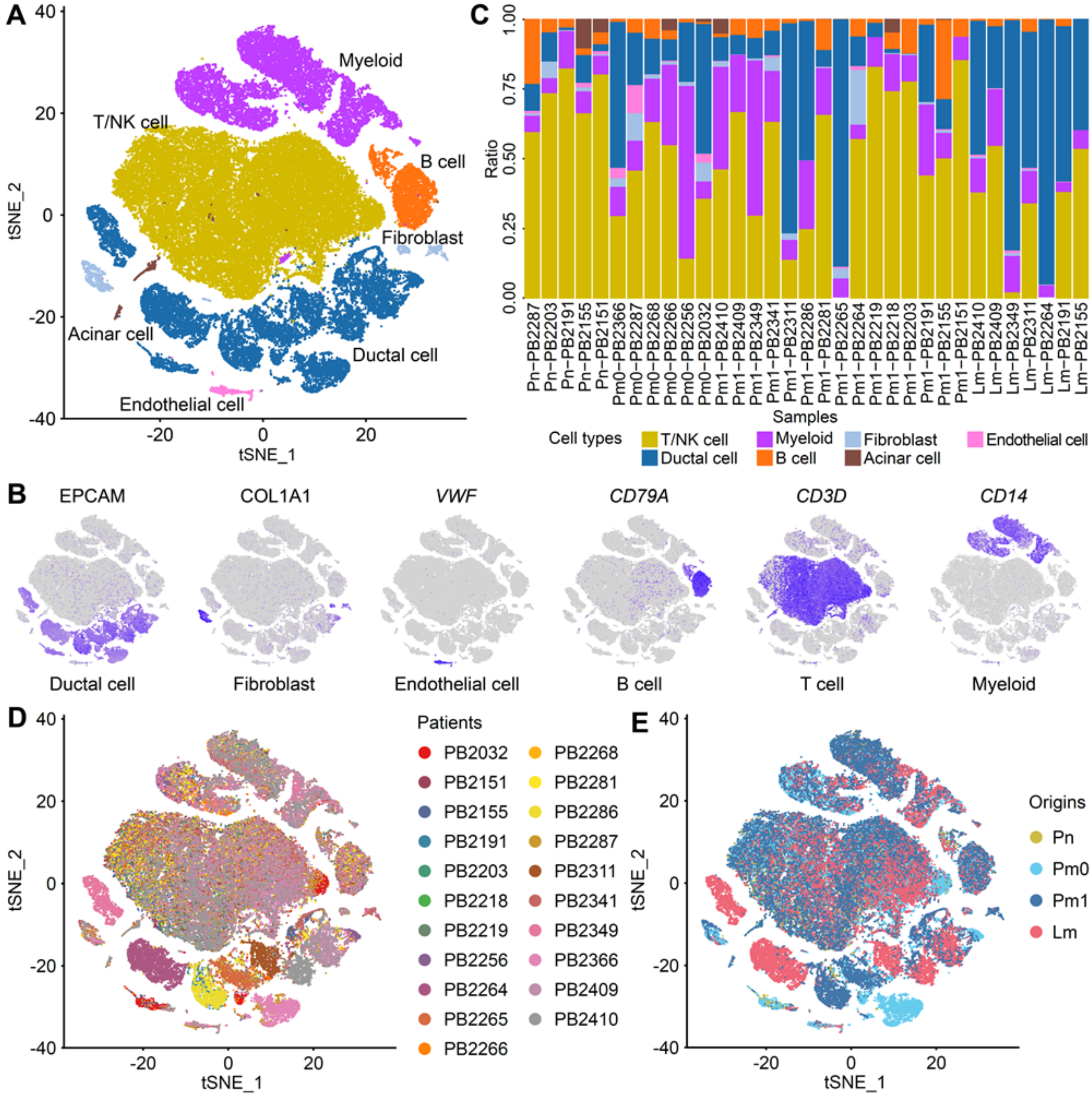

**Fig. S1** Overview of single cells from the primary PDAC mass and matched liver metastasis. **A**, t-SNE projection of 69,204 single cells showing the formation of 21 clusters, including ductal cells, T cells, myeloid cells, fibroblasts, B cells, acinar cells, and endothelial cells. **B**, t-SNE projections displaying the expression of known cell type marker genes. **C**, Bar plot showing the proportion of major cell types in each sample from each patient. **D** and **E**, t-SNE projections of scRNA-seq data isolated from individual PDAC patients. Each dot represents one cell and the colors represent the patients (**D**) and the origins (**E**).

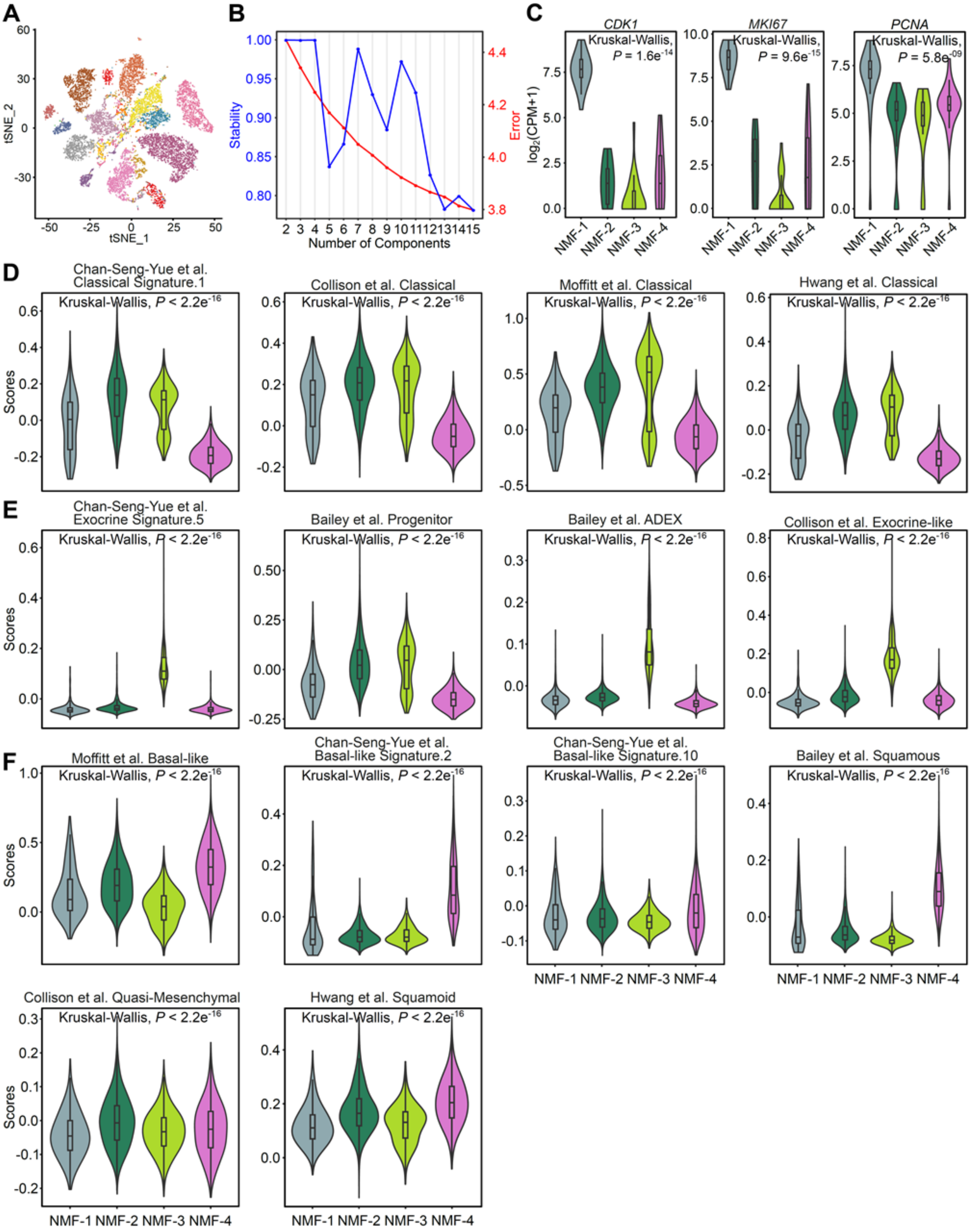
A
tSNE_2
tSNE_1
B
Stability
Error
Number of Components
C
CDK1
MKI67
PCNA
log2(CPM+1)
Kruskal-Wallis, P = 1.6e-14
Kruskal-Wallis, P = 9.6e-15
Kruskal-Wallis, P = 5.8e-09
D
Chan-Seng-Yue et al. Classical Signature.1
Collison et al. Classical
Moffitt et al. Classical
Hwang et al. Classical
E
Chan-Seng-Yue et al. Exocrine Signature.5
Bailey et al. Progenitor
Bailey et al. ADEX
Collison et al. Exocrine-like
F
Moffitt et al. Basal-like
Chan-Seng-Yue et al. Basal-like Signature.2
Chan-Seng-Yue et al. Basal-like Signature.10
Bailey et al. Squamous
Collison et al. Quasi-Mesenchymal
Hwang et al. Squamoid
Kruskal-Wallis, P < 2.2e-16
Scores
NMF-1 NMF-2 NMF-3 NMF-4

**Fig. S2** Molecular subtypes of ductal cells in the primary PDAC mass and matched liver metastasis. **A**, t-SNE projection showing 21 sub-clusters of ductal cells. Each dot is color-coded by the patients. **B**, Stability and error of cNMF solutions for ductal cells with different numbers of programs. **C**, Violin plots showing the expression level of cell cycle related genes for each NMF subtype. **D-F**, Violin plots displaying the scores of signature genes from previously reported classical (**D**), exocrine (**E**), and basal-like (**F**) subtypes.

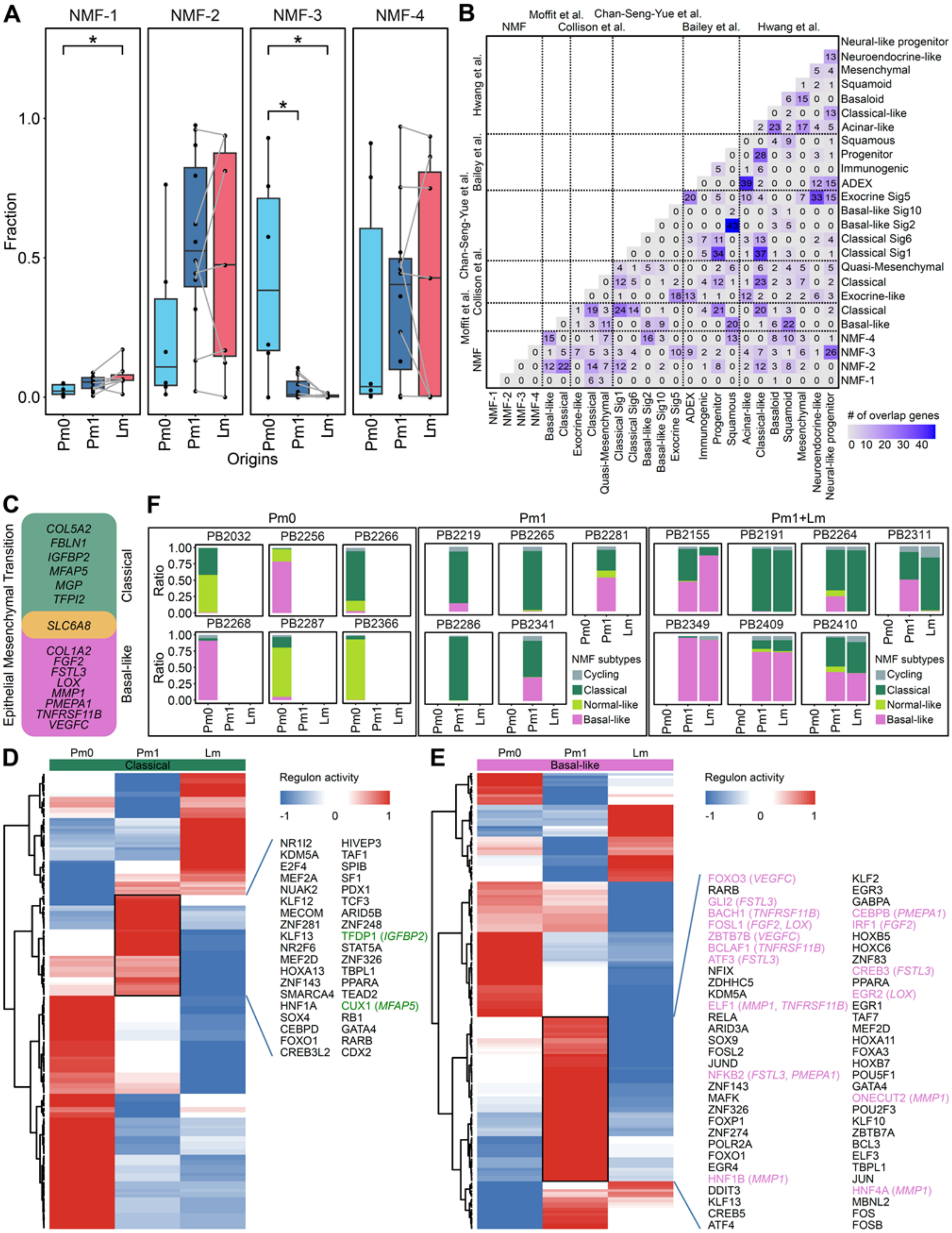

A
NMF-1
NMF-2
NMF-3
NMF-4
Fraction
Origins
Pm0
Pm1
Lm
B
NMF
Moffit et al.
Collison et al.
Chan-Seng-Yue et al.
Bailey et al.
Hwang et al.
Neural-like progenitor
Neuroendocrine-like
Mesenchymal
Squamoid
Basaloid
Classical-like
Acinar-like
Squamous
Progenitor
Immunogenic
ADEX
Exocrine Sig5
Basal-like Sig10
Basal-like Sig2
Classical Sig6
Classical Sig1
Quasi-Mesenchymal
Classical
Exocrine-like
Classical
Basal-like
NMF-4
NMF-3
NMF-2
NMF-1
# of overlap genes
C
Epithelial Mesenchymal Transition
COL5A2
FBLN1
IGFBP2
MFAP5
MGP
TFPI2
Classical
SLC6A8
COL1A2
FGF2
FSTL3
LOX
MMP1
PMEPA1
TNFRSF11B
VEGFC
Basal-like
F
Pm0
Pm1
Pm1+Lm
PB2032
PB2256
PB2266
PB2219
PB2265
PB2281
PB2155
PB2191
PB2264
PB2311
PB2268
PB2287
PB2366
PB2286
PB2341
PB2349
PB2409
PB2410
Ratio
NMF subtypes
Cycling
Classical
Normal-like
Basal-like
D
Classical
Regulon activity
NR1I2
KDM5A
E2F4
MEF2A
NUAK2
KLF12
MECOM
ZNF281
KLF13
NR2F6
MEF2D
HOXA13
ZNF143
SMARCA4
HNF1A
SOX4
CEBPD
FOXO1
CREB3L2
HIVEP3
TAF1
SPIB
SF1
PDX1
TCF3
ARID5B
ZNF248
TFDP1 (IGFBP2)
STAT5A
ZNF326
TBPL1
PPARA
TEAD2
CUX1 (MFAP5)
RB1
GATA4
RARB
CDX2
E
Basal-like
Regulon activity
FOXO3 (VEGFC)
RARB
GLI2 (FSTL3)
BACH1 (TNFRSF11B)
FOSL1 (FGF2, LOX)
ZBTB7B (VEGFC)
BCLAF1 (TNFRSF11B)
ATF3 (FSTL3)
NFIX
ZDHHC5
KDM5A
ELF1 (MMP1, TNFRSF11B)
RELA
ARID3A
SOX9
FOSL2
JUND
NFKB2 (FSTL3, PMEPA1)
ZNF143
MAFK
ZNF326
FOXP1
ZNF274
POLR2A
FOXO1
EGR4
HNF1B (MMP1)
DDIT3
KLF13
CREB5
ATF4
KLF2
EGR3
GABPA
CEBPB (PMEPA1)
IRF1 (FGF2)
HOXB5
HOXC6
ZNF83
CREB3 (FSTL3)
PPARA
EGR2 (LOX)
EGR1
TAF7
MEF2D
HOXA11
FOXA3
HOXB7
POU5F1
GATA4
ONECUT2 (MMP1)
POU2F3
KLF10
ZBTB7A
BCL3
ELF3
TBPL1
JUN
HNF4A (MMP1)
MBNL2
FOS
FOSB

**Fig. S3** Relationship between PDAC subtype and patient prognosis. **A**, Box plots showing the percentage differences in NMF subtypes among origins (two-sided Wilcoxon rank sum test: *$P < 0.05$, **$P < 0.01$, ***$P < 0.001$). Samples from the same patients were connected by solid lines. **B**, Heatmap showing the shared signature genes between NMF subtypes and previously reported subtype signature genes. **C**, Venn diagram illustrating the similarity of EMT genes related to classical or basal-like subtype. **D** and **E**, Heatmaps showing Pm1-specifically activated TFs in classical and basal-like PDAC subtypes. TFs regulating the PDAC subtype specific EMT genes in Pm1 are colored to match the subtypes. **F**, Bar plots showing the fraction of four NMF subtypes in each sample, and each color indicated the subtype.

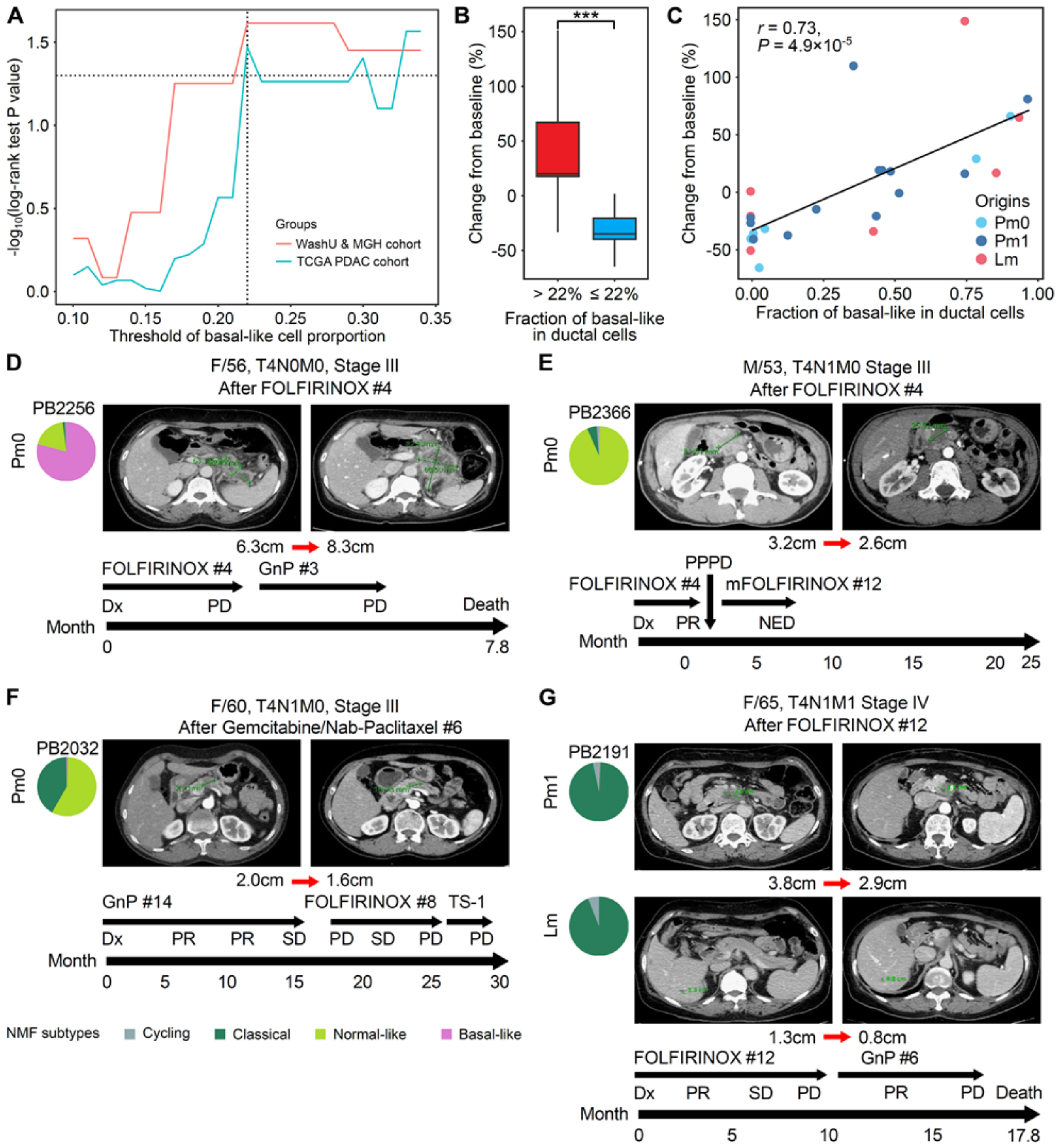

A
-log10(log-rank test P value)
Threshold of basal-like cell prorportion
Groups
WashU & MGH cohort
TCGA PDAC cohort
B
***
Change from baseline (%)
> 22% ≤ 22%
Fraction of basal-like in ductal cells
C
r = 0.73, P = 4.9×10-5
Change from baseline (%)
Fraction of basal-like in ductal cells
Origins
Pm0
Pm1
Lm
D
F/56, T4N0M0, Stage III
After FOLFIRINOX #4
PB2256
Pm0
6.3cm → 8.3cm
FOLFIRINOX #4
GnP #3
Dx PD PD Death
Month
0 7.8
E
M/53, T4N1M0 Stage III
After FOLFIRINOX #4
PB2366
Pm0
3.2cm → 2.6cm
PPPD
FOLFIRINOX #4
mFOLFIRINOX #12
Dx PR NED
Month
0 5 10 15 20 25
F
F/60, T4N1M0, Stage III
After Gemcitabine/Nab-Paclitaxel #6
PB2032
Pm0
2.0cm → 1.6cm
GnP #14
FOLFIRINOX #8
TS-1
Dx PR PR SD PD SD PD PD
Month
0 5 10 15 20 25 30
G
F/65, T4N1M1 Stage IV
After FOLFIRINOX #12
PB2191
Pm1
3.8cm → 2.9cm
Lm
1.3cm → 0.8cm
FOLFIRINOX #12
GnP #6
Dx PR SD PD PR PD Death
Month
0 5 10 15 17.8
NMF subtypes
Cycling
Classical
Normal-like
Basal-like

**Fig. S4** An investigation of treatment courses for patients with different NMF subtypes. **A**, Line plot showing Kaplan-Meier (log-rank) test *P* values (horizontal dotted line for $P$=0.05) when dividing samples of the TCGA PDAC cohort (bulk RNA-seq, $N$=148) and WashU and MGH cohorts (scRNA-seq, $N$=25) into two groups based on a range of basal-like cell proportions (10~35%). The minimum basal-like cell proportion exhibiting a statistical association with survival in both cohorts is 22% (vertical dotted line). **B**, Boxplot showing the distribution of change in the sum of the target lesions (RECIST v1.1) within two groups (two-sided Wilcoxon rank sum test: *$P < 0.05$, **$P < 0.01$, ***$P < 0.001$). **C**, Scatter plot displaying the Pearson correlation between change in the sum of the target lesions (RECIST v1.1) and the fraction of basal-like in ductal cells. **D-G**, The proportion of PDAC NMF subtypes and CT scan images before and after chemotherapy of PDAC patients PB2256 (**D**), PB2366 (**E**), PB2032 (**F**), and PB2191 (**G**).

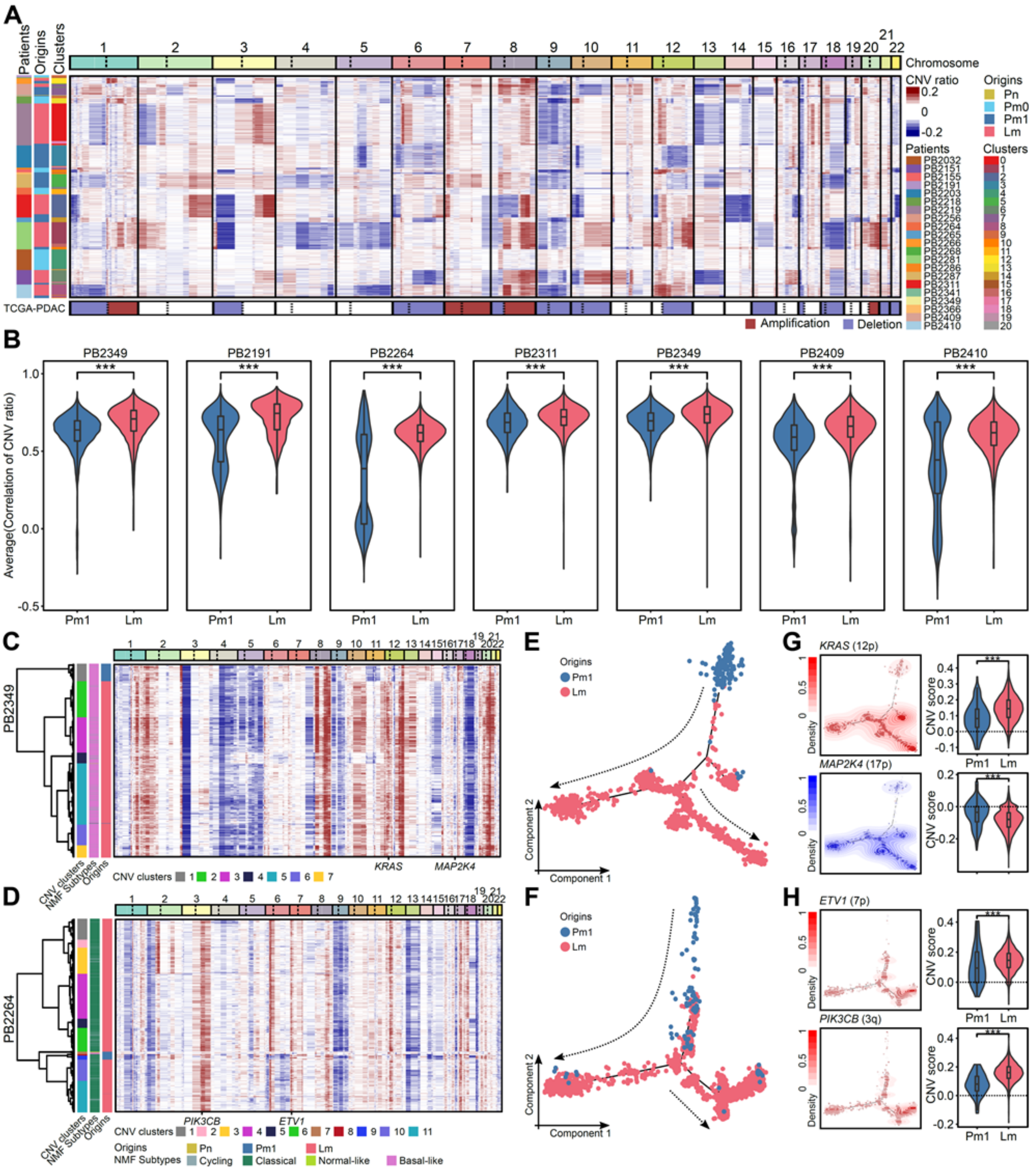
A
Patients
Origins
Clusters
Chromosome
1 2 3 4 5 6 7 8 9 10 11 12 13 14 15 16 17 18 19 20 21 22
CNV ratio
0.2
0
-0.2
Origins
Pn
Pm0
Pm1
Lm
Patients
PB2032
PB2151
PB2155
PB2191
PB2203
PB2218
PB2219
PB2256
PB2264
PB2265
PB2266
PB2268
PB2281
PB2286
PB2287
PB2311
PB2341
PB2349
PB2366
PB2409
PB2410
Clusters
0 1 2 3 4 5 6 7 8 9 10 11 12 13 14 15 16 17 18 19 20
TCGA-PDAC
Amplification
Deletion
B
PB2349
PB2191
PB2264
PB2311
PB2349
PB2409
PB2410
***
Average(Correlation of CNV ratio)
1.0
0.5
0.0
-0.5
Pm1
Lm
C
PB2349
KRAS
MAP2K4
CNV clusters 1 2 3 4 5 6 7
CNV clusters
NMF Subtypes
Origins
D
PB2264
PIK3CB
ETV1
CNV clusters 1 2 3 4 5 6 7 8 9 10 11
Origins Pn Pm1 Lm
NMF Subtypes Cycling Classical Normal-like Basal-like
E
Origins
Pm1
Lm
Component 1
Component 2
F
Origins
Pm1
Lm
Component 1
Component 2
G
KRAS (12p)
MAP2K4 (17p)
Density
CNV score
Pm1
Lm
***
H
ETV1 (7p)
PIK3CB (3q)
Density
CNV score
Pm1
Lm
***

**Fig. S5** Clonal evolution of ductal cells in PDAC progression and metastasis. **A**, Heatmap showing CNVs profiles of ductal cells from primary PDAC masses and matched liver metastases. Each cell is sorted by patient and sample (left sidebar). The bottom sidebar shows the CNV profiles at the genomic level of TCGA PDAC patients. **B**, Violin plots showing the distribution of mean CNV correlation coefficients among malignant ductal cells within origin by patient (two-sided Wilcoxon rank sum test: $^*P < 0.05$, $^{**}P < 0.01$, $^{***}P < 0.001$). **C** and **D**, Hierarchical clustering of CNV profiles in individual patients PB2349 (**C**) and PB2264 (**D**). **E** and **F**, Unsupervised transcriptional trajectories of ductal cells in individual patients PB2349 (**E**) and PB2264 (**F**) colored by sample origin. Trajectory directions were indicated by arrows. **G** and **H**, Dots on trajectory projections (left) were colored by copy number scores at the cellular level and overlaid with contour plots of cells with the strongest copy number variation for known cancer-associated genes in individual patients PB2349 (**G**) and PB2264 (**H**). Violin plots (right) showed copy number scores of genes by origin (two-sided Wilcoxon rank sum test: $^*P < 0.05$, $^{**}P < 0.01$, $^{***}P < 0.001$).

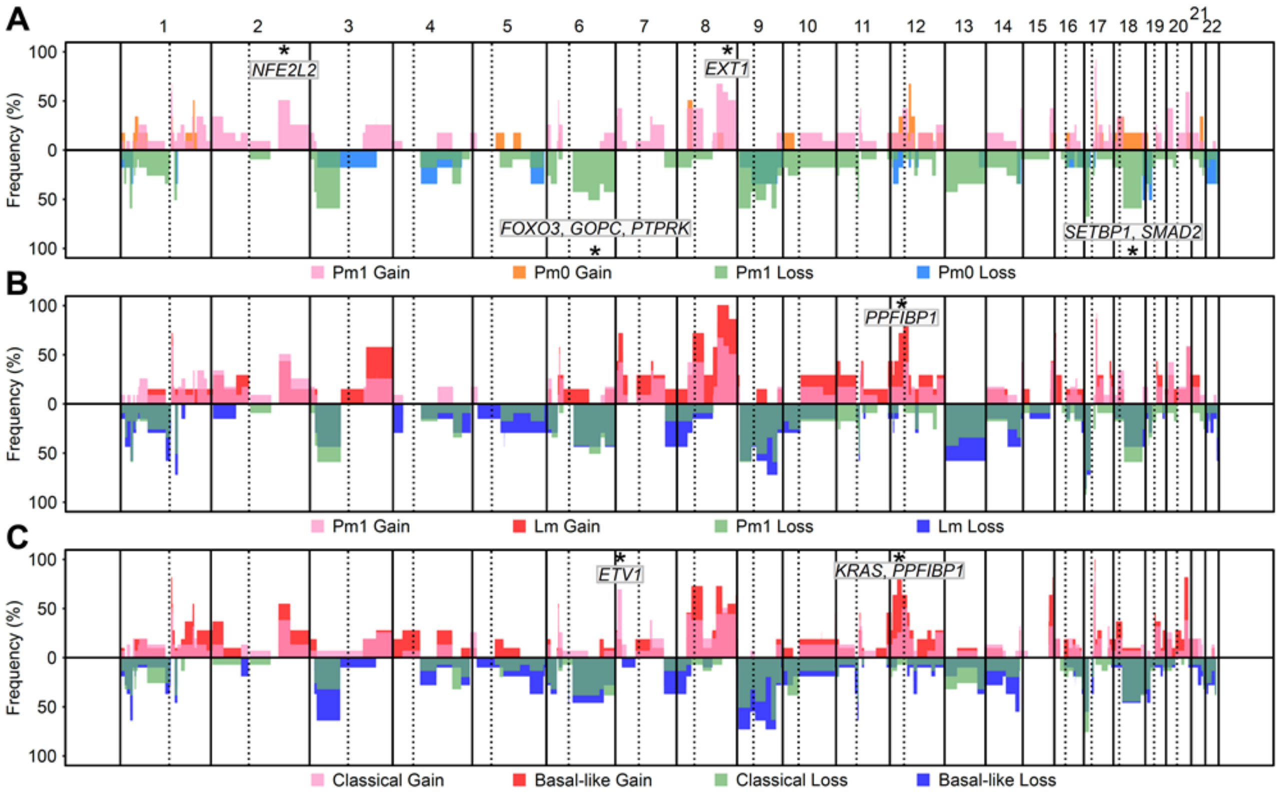


**Fig. S6** Clonal evolution during PDAC progression. **A**-**C**, Histograms showing the frequency of CNV gains (pink and red) or losses (green and blue) according to origins (**A**, Pm0 versus Pm1; **B**, Pm1 versus Lm) or PDAC subtypes (**C**, classical versus basal-like). Asterisks indicated regions showing significant differences through Fisher's exact test ($P < 0.05$).

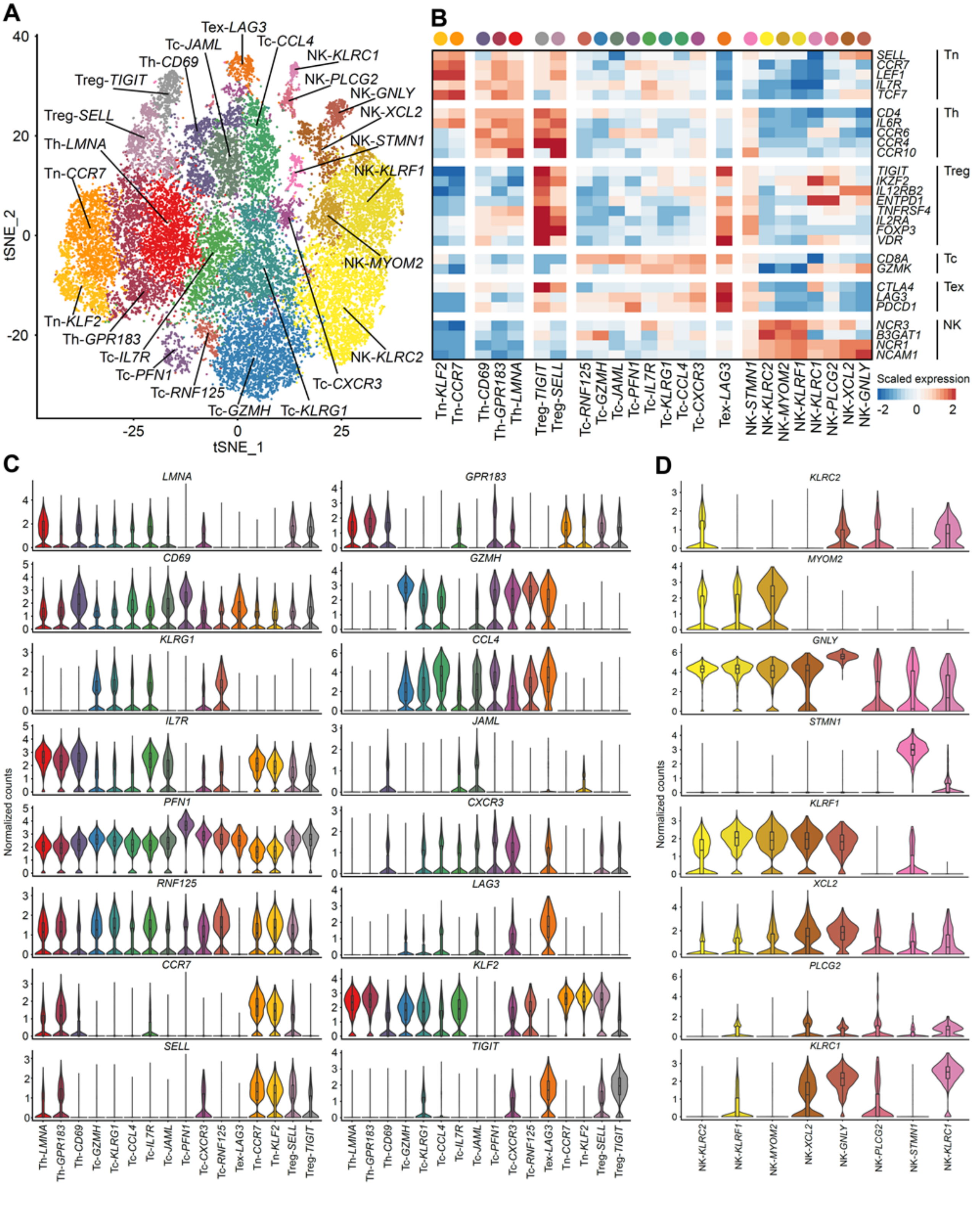

A
B
C
D
tSNE_1
tSNE_2
Tex-LAG3
Tc-CCL4
Tc-JAML
Th-CD69
Treg-TIGIT
Treg-SELL
Th-LMNA
Tn-CCR7
Tn-KLF2
Th-GPR183
Tc-IL7R
Tc-PFN1
Tc-RNF125
Tc-GZMH
Tc-KLRG1
Tc-CXCR3
NK-KLRC1
NK-PLCG2
NK-GNLY
NK-XCL2
NK-STMN1
NK-KLRF1
NK-MYOM2
NK-KLRC2
Tn
Th
Treg
Tc
Tex
NK
Scaled expression
Normalized counts

**Fig. S7** T/NK cells subclusters in the primary PDAC mass and matched liver metastasis. **A**, t-SNE projection showing 24 subclusters of T/NK cells. Each dot is color-coded by cluster. **B**, Heatmap showing the expression level of cell type marker genes in each T/NK cell subcluster. **C** and **D**, Expression level of cell type markers for T (**C**) and NK (**D**) cell subclusters.

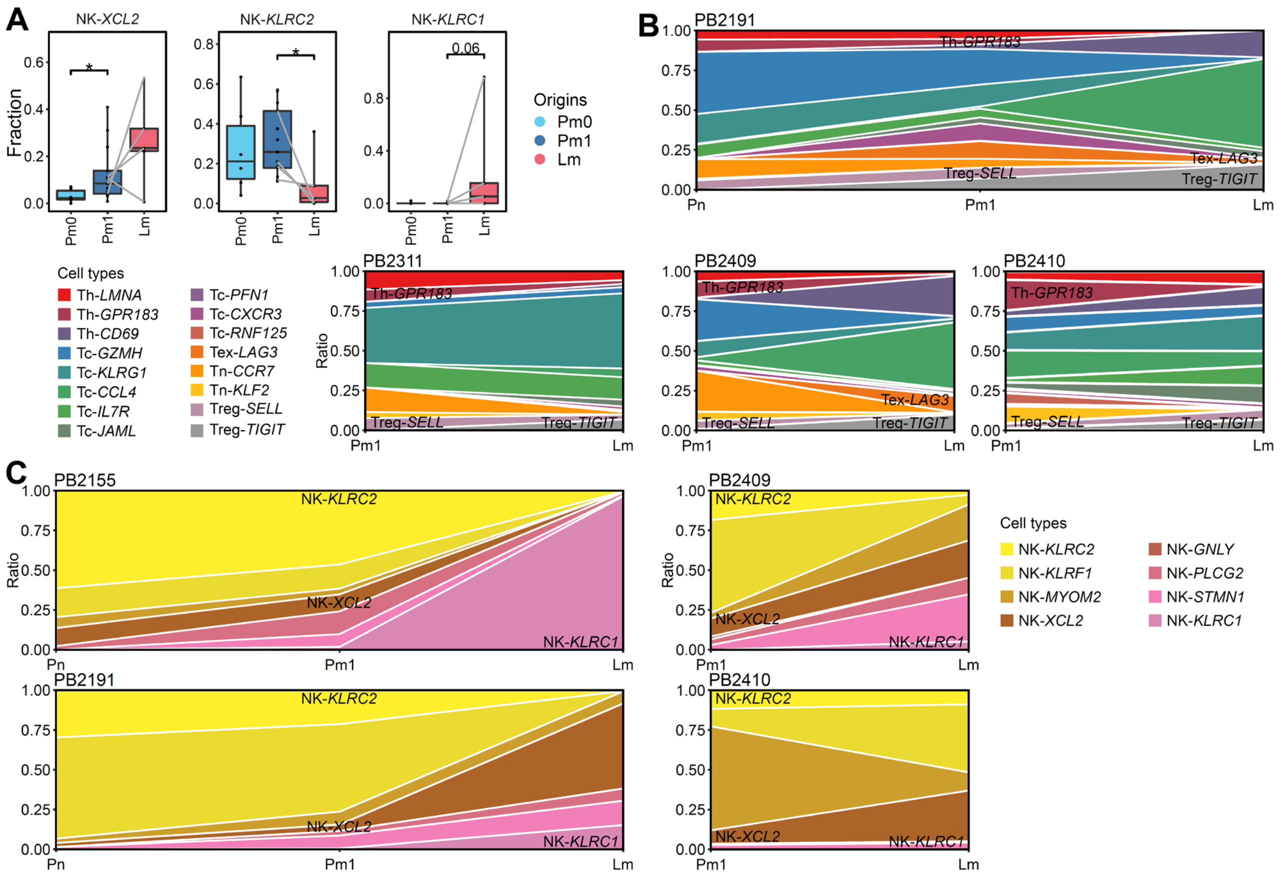


**Fig. S8** Variations in the composition of T/NK subclusters according to patient origin. **A**, Box plots indicating the percentage differences in T/NK cell subclusters among origins (two-sided Wilcoxon rank sum test: *$P < 0.05$, **$P < 0.01$, ***$P < 0.001$). Samples from the same patients were connected by solid lines. **B** and **C**, Area plots displaying the changes in T cell (**B**) and NK (**C**) subcluster composition by origin for each patient.

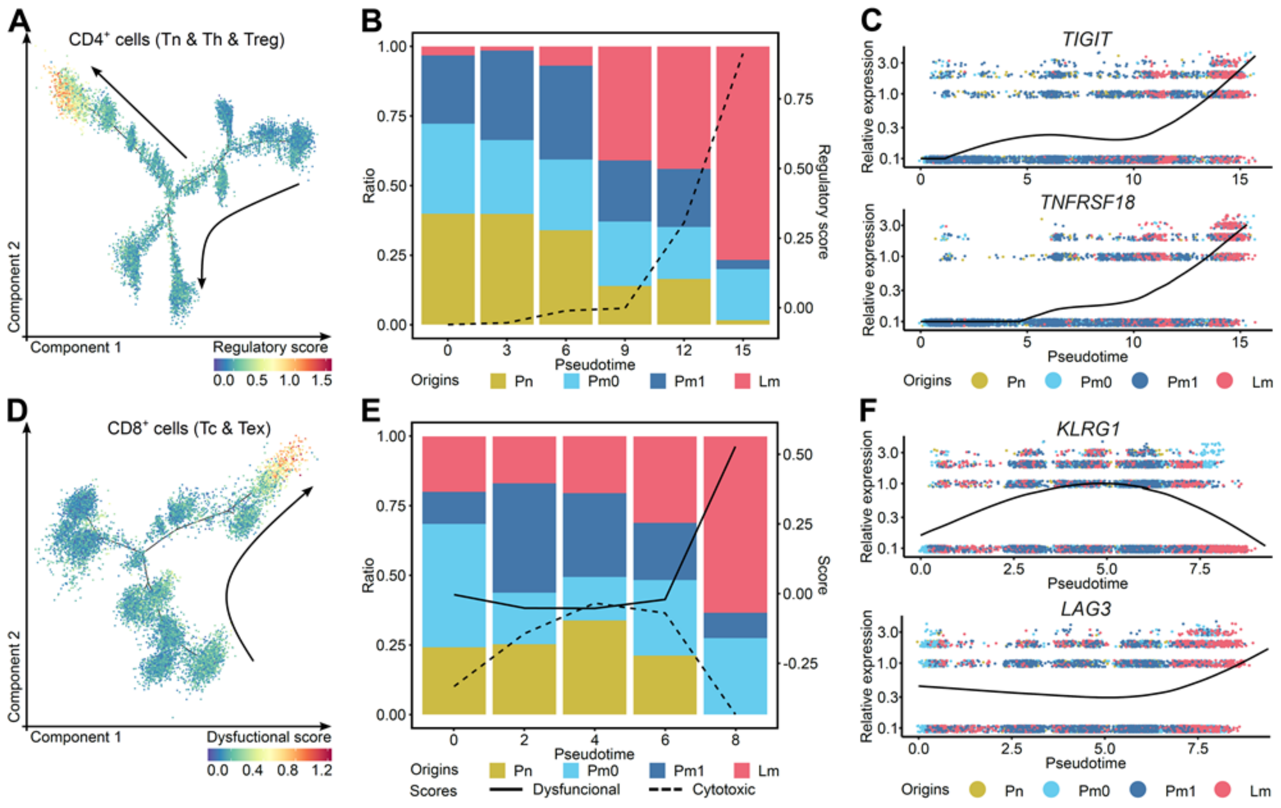
A
CD4⁺ cells (Tn & Th & Treg)
Component 1
Component 2
Regulatory score
0.0 0.5 1.0 1.5
B
Ratio
Regulatory score
Pseudotime
Origins Pn Pm0 Pm1 Lm
C
TIGIT
TNFRSF18
Relative expression
Pseudotime
Origins Pn Pm0 Pm1 Lm
D
CD8⁺ cells (Tc & Tex)
Component 1
Component 2
Dysfunctional score
0.0 0.4 0.8 1.2
E
Ratio
Score
Pseudotime
Origins Pn Pm0 Pm1 Lm
Scores Dysfuncional Cytotoxic
F
KLRG1
LAG3
Relative expression
Pseudotime
Origins Pn Pm0 Pm1 Lm

**Fig. S9** Lineage analysis of T cells in PDAC progression and metastasis. **A**, Unsupervised transcriptional trajectory of $CD4^+$ T cells (Tn, Th and Treg) in primary PDAC and liver metastasis. The color of each dot represents a regulatory score. Trajectory directions were indicated by arrows. **B**, Bar plot showing the ratio of origins at each pseudotime interval, and each color represents an origin. The dotted line indicates the average regulatory score for each pseudotime interval. **C**, Relative expression of representative regulatory genes, *TIGIT* and *TNFRSF18*, along pseudotime, and each dot is colored by origins. **D**, Unsupervised transcriptional trajectory of $CD8^+$ T cells (Tc and Tex) in primary PDAC and liver metastasis. The color of each dot represents a dysfunctional score. Trajectory directions were indicated by arrows. **E**, Bar plot showing the ratio of origins at each pseudotime interval, and each color represents an origin. Mean dysfunctional and cytotoxic scores for pseudotime intervals were illustrated by solid and dotted lines, respectively. **F**, Relative expression of representative cytotoxic (*KLRG1*) and dysfunctional (*LAG3*) genes along pseudotime, and each dot is colored by origins.

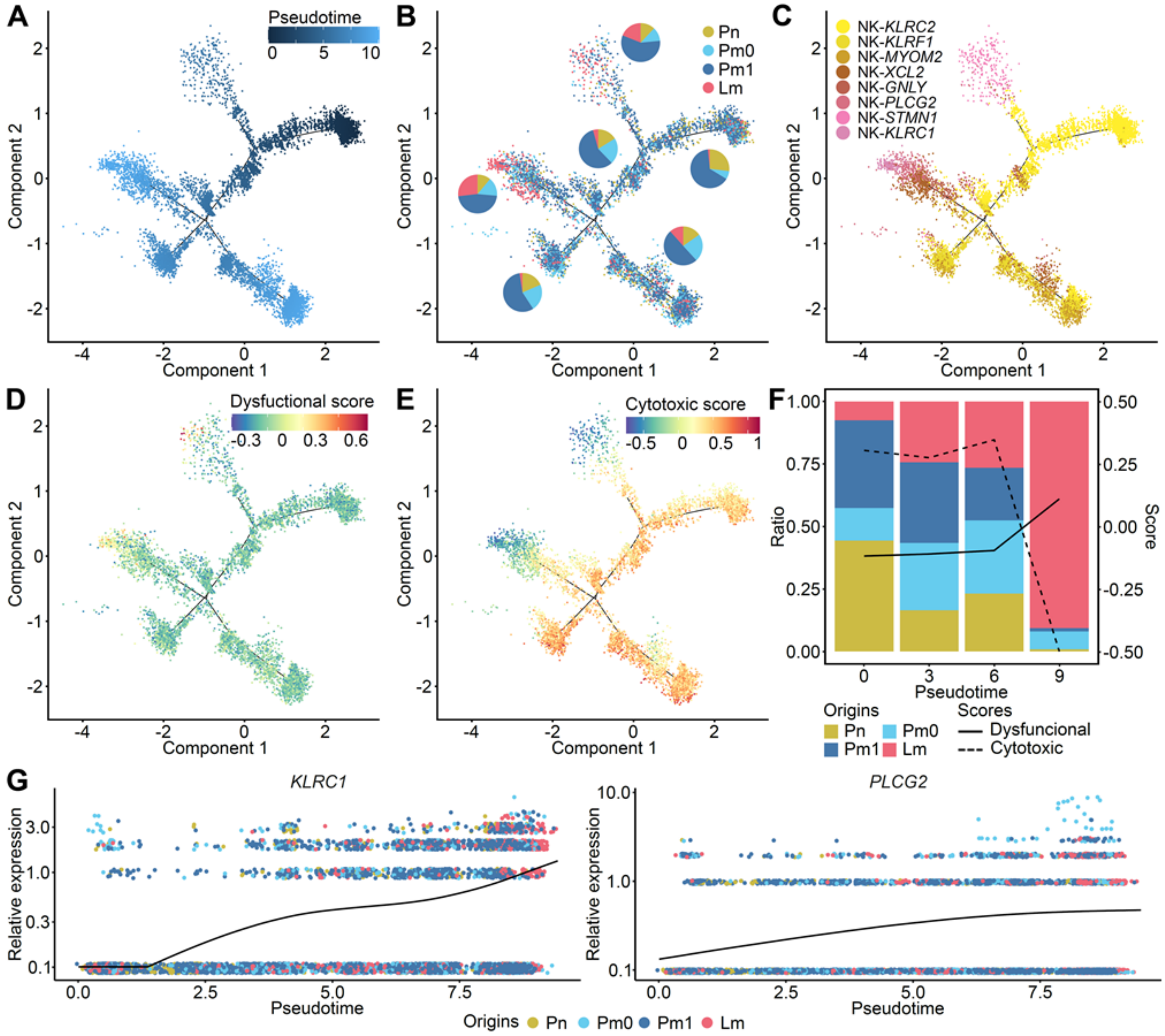


**Fig. S10** Lineage analysis of NK cells in PDAC progression and metastasis. **A**-**E**, Unsupervised transcriptional trajectory of NK cell in primary PDAC mass and liver metastasis biopsy. The color of each dot is indicated by pseudotime (**A**), origins (**B**), NK cell subtypes (**C**), dysfunctional score (**D**), and cytotoxicity score (**E**). **F**, Bar plot showing the ratio of origins at each pseudotime interval, and each color represents an origin. Mean dysfunctional and cytotoxic scores for pseudotime intervals were illustrated by solid and dotted lines, respectively. **G**, Relative expression of representative dysfunctional (*KLRC1* and *PLCG2*) genes across pseudotime, and each dot is colored by origins.

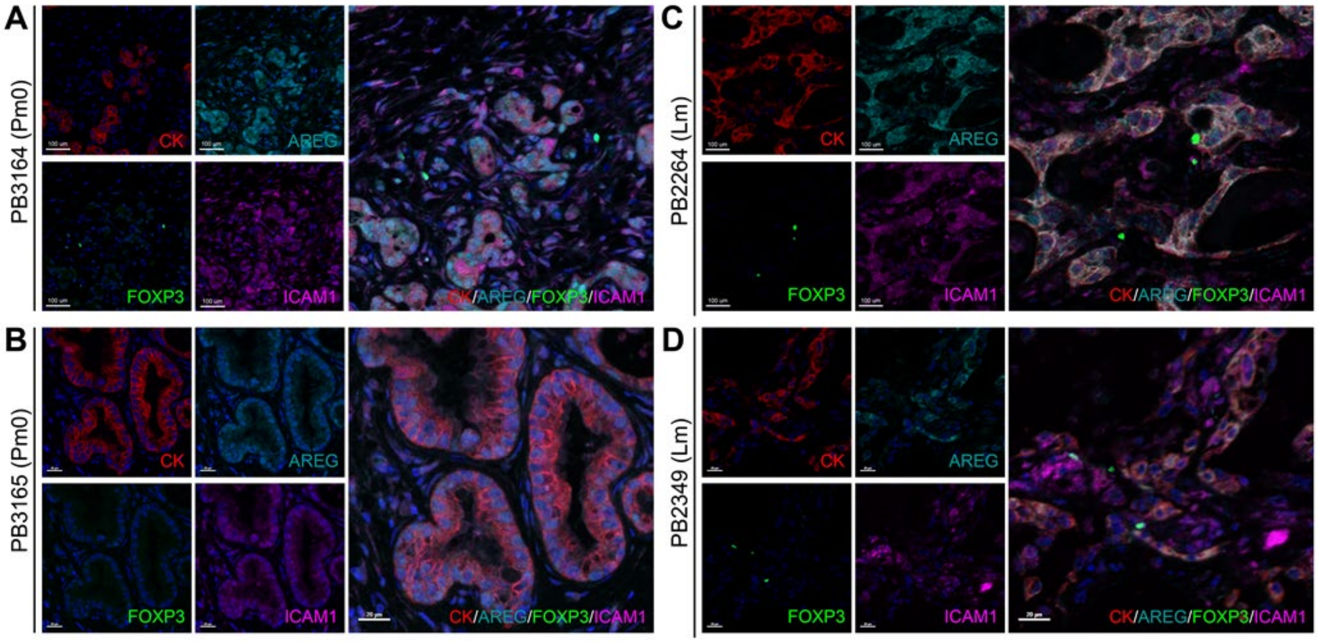


**Fig. S11** Receptor-ligand interactions between ductal cancer cells and regulatory T cells (Tregs) are examined in primary pancreas (Pm0, **A** and **B**) and liver metastasis (Lm, **C** and **D**) tissues by multiplex immunohistochemistry. AREG (cyan)-ICAM1 (magenta); Ductal cancer cells are stained with pan-cytokeratin (CK, red), and Tregs are stained with FOXP3 (green). Nuclei were counterstained with DAPI (blue): scale bar, 20 μm.

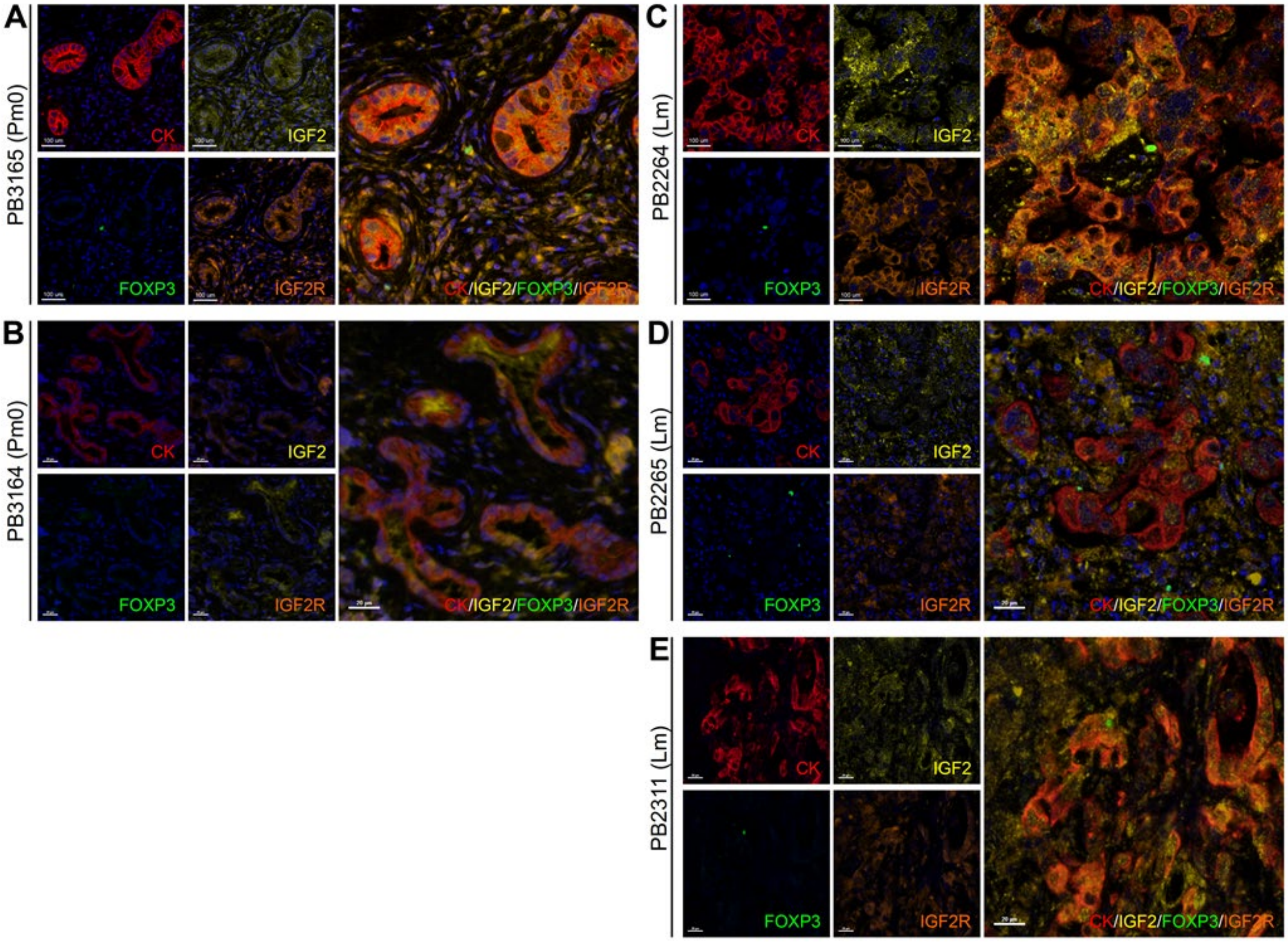


**Fig. S12** Receptor-ligand interactions between ductal cancer cells and regulatory T cells (Tregs) are examined in primary pancreas (Pm0, **A** and **B**) and liver metastasis (Lm, **C**-**E**) tissues by multiplex immunohistochemistry. IGF2 (yellow)-IGF2R (orange); Ductal cancer cells are stained with pan-cytokeratin (CK, red), and Tregs are stained with FOXP3 (green). Nuclei were counterstained with DAPI (blue): scale bar, 20 μm.

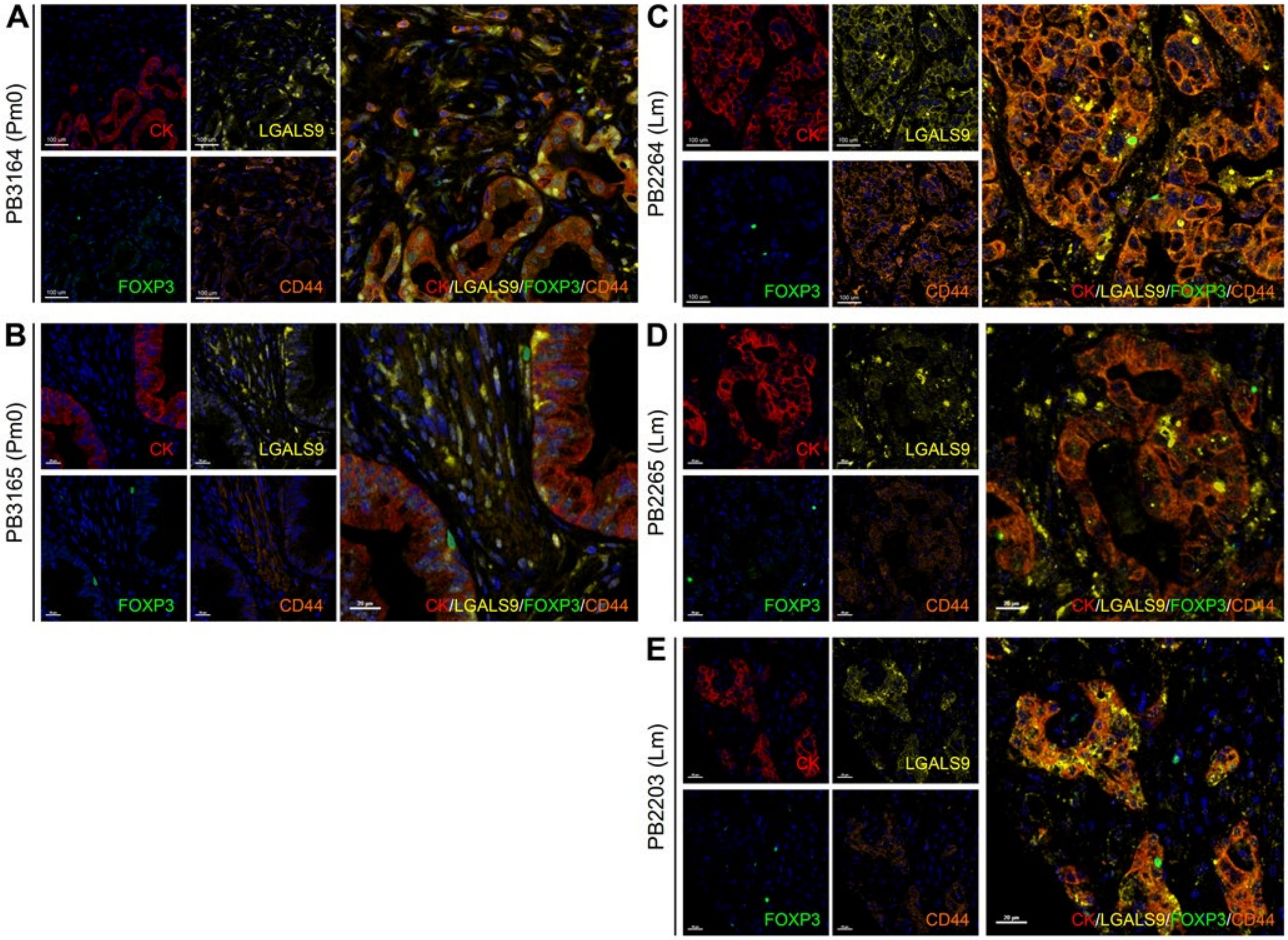


**Fig. S13** Receptor-ligand interactions between ductal cancer cells and regulatory T cells (Tregs) are examined in primary pancreas (Pm0, **A**-**C**) and liver metastasis (Lm, **D**-**F**) tissues by multiplex immunohistochemistry. LGALS9 (yellow)-CD44 (orange); Ductal cancer cells are stained with pan-cytokeratin (CK, red), and Tregs are stained with FOXP3 (green). Nuclei were counterstained with DAPI (blue): scale bar, 20 μm.

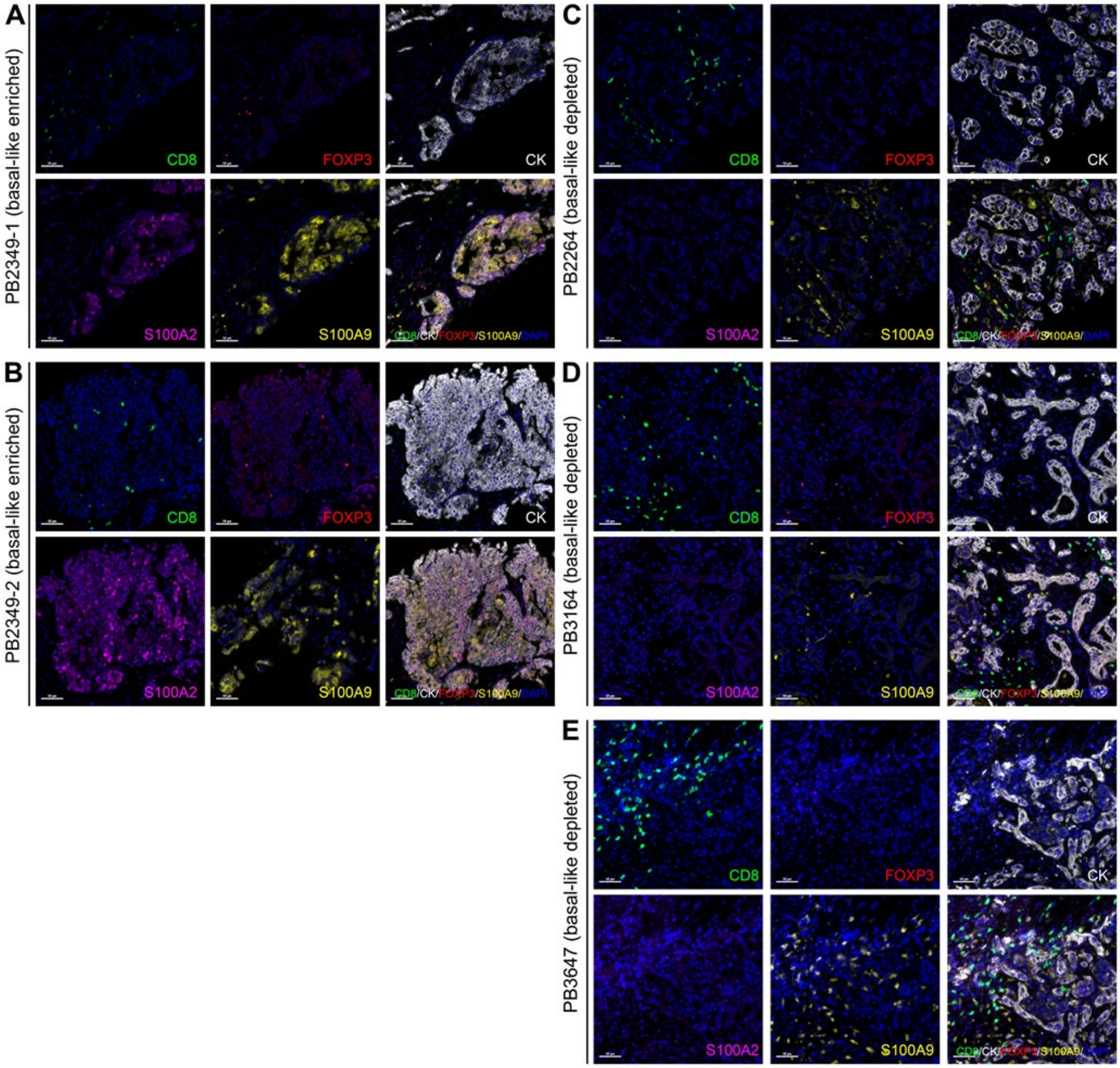


**Fig. S14** T cell distribution and S100A9 expression in basal-like and classical PDAC tissues. Multiplex IHC showing the expression of S100A9 (yellow) and the distribution of T cells in basal-like enriched (**A** and **B**) and depleted (**C**-**D**) PDAC tissues. CD8 (green) for cytotoxic T cells, FOXP3 (red) for regulatory T cells, pan-cytokeratin (CK, white) for ductal cancer cells, S100A2 (magenta) for basal-like ductal cells and DAPI (blue) for nuclei were co-stained. Scale bar, 50 μm.

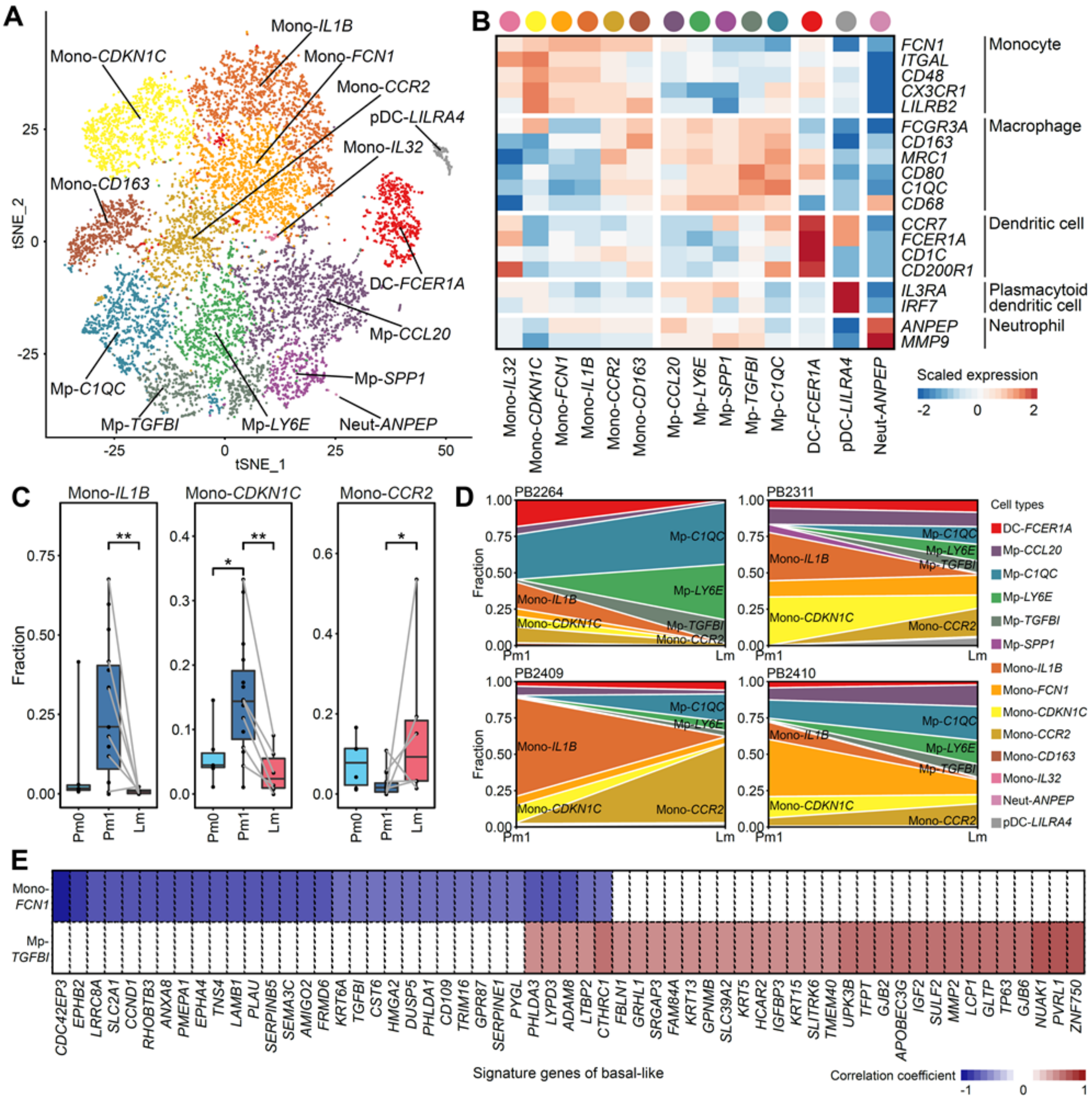

A
Mono-IL1B
Mono-CDKN1C
Mono-FCN1
Mono-CCR2
pDC-LILRA4
Mono-IL32
Mono-CD163
DC-FCER1A
Mp-CCL20
Mp-SPP1
Neut-ANPEP
Mp-C1QC
Mp-TGFBI
Mp-LY6E
tSNE_1
tSNE_2
B
FCN1
ITGAL
CD48
CX3CR1
LILRB2
Monocyte
FCGR3A
CD163
MRC1
CD80
C1QC
CD68
Macrophage
CCR7
FCER1A
CD1C
CD200R1
Dendritic cell
IL3RA
IRF7
Plasmacytoid dendritic cell
ANPEP
MMP9
Neutrophil
Scaled expression
C
Fraction
Pm0
Pm1
Lm
D
PB2264
PB2311
PB2409
PB2410
Fraction
Cell types
DC-FCER1A
Mp-CCL20
Mp-C1QC
Mp-LY6E
Mp-TGFBI
Mp-SPP1
Mono-IL1B
Mono-FCN1
Mono-CDKN1C
Mono-CCR2
Mono-CD163
Mono-IL32
Neut-ANPEP
pDC-LILRA4
E
Mono-FCN1
Mp-TGFBI
CDC42EP3 EPHB2 LRRC8A SLC2A1 CCND1 RHOBTB3 ANXA8 PMEPA1 EPHA4 TNS4 LAMB1 PLAU SERPINB5 SEMA3C AMIGO2 FRMD6 KRT6A TGFBI CST6 HMGA2 DUSP5 PHLDA1 CD109 TRIM16 GPR87 SERPINE1 PYGL PHLDA3 LYPD3 ADAM8 LTBP2 CTHRC1 FBLN1 GRHL1 SRGAP3 FAM84A KRT13 GPNMB SLC39A2 KRT5 HCAR2 IGFBP3 KRT15 SLITRK6 TMEM40 UPK3B TFPT GJB2 APOBEC3G IGF2 SULF2 MMP2 LCP1 GLTP TP63 GJB6 NUAK1 PVRL1 ZNF750
Signature genes of basal-like
Correlation coefficient
-1
0
1

**Fig. S15** Myeloid subclusters in the primary PDAC mass and matched liver metastasis. **A**, t-SNE projections showing 14 subclusters of myeloid cells. Each point is color-coded by a cluster. **B**, Heatmap showing the expression level of cell type markers in each myeloid subcluster. **C**, Box plots indicating the percentage differences in myeloid subclusters among origins (two-sided Wilcoxon rank sum test: *$P < 0.05$, **$P < 0.01$, ***$P < 0.001$). Samples from the same patients were connected by solid lines. **D**, Area plots showing the change in the composition of the myeloid subclusters by origin for each patient. **E**, Pearson correlation between the expression level of basal-like signature genes in the ductal cells and the fractions of Mp-*TGFBI* and Mono-*FCN1* in myeloid cells.

## REFERENCES


1. Krieger TG, Le Blanc S, Jabs J, Ten FW, Ishaque N, Jechow K, Debnath O, Leonhardt CS, Giri A, Eils R, et al: **Single-cell analysis of patient-derived PDAC organoids reveals cell state heterogeneity and a conserved developmental hierarchy.** *Nat Commun* 2021, **12:**5826.
2. Chan-Seng-Yue M, Kim JC, Wilson GW, Ng K, Figueroa EF, O'Kane GM, Connor AA, Denroche RE, Grant RC, McLeod J, et al: **Transcription phenotypes of pancreatic cancer are driven by genomic events during tumor evolution.** *Nat Genet* 2020, **52:**231-240.
3. Collisson EA, Sadanandam A, Olson P, Gibb WJ, Truitt M, Gu S, Cooc J, Weinkle J, Kim GE, Jakkula L, et al: **Subtypes of pancreatic ductal adenocarcinoma and their differing responses to therapy.** *Nat Med* 2011, **17:**500-503.
4. Moffitt RA, Marayati R, Flate EL, Volmar KE, Loeza SG, Hoadley KA, Rashid NU, Williams LA, Eaton SC, Chung AH, et al: **Virtual microdissection identifies distinct tumor- and stroma-specific subtypes of pancreatic ductal adenocarcinoma.** *Nat Genet* 2015, **47:**1168-1178.
5. Bailey P, Chang DK, Nones K, Johns AL, Patch AM, Gingras MC, Miller DK, Christ AN, Bruxner TJ, Quinn MC, et al: **Genomic analyses identify molecular subtypes of pancreatic cancer.** *Nature* 2016, **531:**47-52.
6. Ko SE, Lee MW, Lim HK, Min JH, Cha DI, Kang TW, Song KD, Kim MJ, Rhim H: **The semi-erect position for better visualization of subphrenic hepatocellular carcinoma during ultrasonography examinations.** *Ultrasonography* 2021, **40:**274-280.
7. Lee MW: **Fusion imaging of real-time ultrasonography with CT or MRI for hepatic intervention.** *Ultrasonography* 2014, **33:**227-239.
8. Dobin A, Davis CA, Schlesinger F, Drenkow J, Zaleski C, Jha S, Batut P, Chaisson M, Gingeras TR: **STAR: ultrafast universal RNA-seq aligner.** *Bioinformatics* 2013, **29:**15-21.
9. Butler A, Hoffman P, Smibert P, Papalexi E, Satija R: **Integrating single-cell transcriptomic data across different conditions, technologies, and species.** *Nat Biotechnol* 2018, **36:**411-420.
10. Aran D, Looney AP, Liu L, Wu E, Fong V, Hsu A, Chak S, Naikawadi RP, Wolters PJ, Abate AR, et al: **Reference-based analysis of lung single-cell sequencing reveals a transitional profibrotic macrophage.** *Nat Immunol* 2019, **20:**163-172.
11. Vieira Braga FA, Kar G, Berg M, Carpaij OA, Polanski K, Simon LM, Brouwer S, Gomes T, Hesse L, Jiang J, et al: **A cellular census of human lungs identifies novel cell states in health and in asthma.** *Nat Med* 2019, **25:**1153-1163.
12. Zhou DC, Jayasinghe RG, Chen SQ, Herndon JM, Iglesia MD, Navale P, Wendl MC, Caravan W, Sato K, Storrs E, et al: **Spatially restricted drivers and transitional cell populations cooperate with the microenvironment in untreated and chemo-resistant pancreatic cancer.** *Nature Genetics* 2022, **54**.
13. Cable DM, Murray E, Zou LLS, Goeva A, Macosko EZ, Chen F, Irizarry RA: **Robust decomposition of cell type mixtures in spatial transcriptomics.** *Nature Biotechnology* 2022, **40:**517-+.
14. Young MD, Behjati S: **SoupX removes ambient RNA contamination from droplet-based single-cell RNA sequencing data.** *Gigascience* 2020, **9**.
15. Wolock SL, Lopez R, Klein AM: **Scrublet: Computational Identification of Cell Doublets in Single-Cell Transcriptomic Data.** *Cell Syst* 2019, **8:**281-291 e289.
16. Kotliar D, Veres A, Nagy MA, Tabrizi S, Hodis E, Melton DA, Sabeti PC: **Identifying gene expression programs of cell-type identity and cellular activity with single-cell RNA-Seq.** *Elife* 2019, **8**.

17. Gao R, Bai S, Henderson YC, Lin Y, Schalck A, Yan Y, Kumar T, Hu M, Sei E, Davis A, et al: **Delineating copy number and clonal substructure in human tumors from single-cell transcriptomes.** *Nat Biotechnol* 2021, **39:**599-608.
18. Li H, van der Leun AM, Yofe I, Lubling Y, Gelbard-Solodkin D, van Akkooi ACJ, van den Braber M, Rozeman EA, Haanen J, Blank CU, et al: **Dysfunctional CD8 T Cells Form a Proliferative, Dynamically Regulated Compartment within Human Melanoma.** *Cell* 2019, **176:**775-789 e718.
19. Kuleshov MV, Jones MR, Rouillard AD, Fernandez NF, Duan Q, Wang Z, Koplev S, Jenkins SL, Jagodnik KM, Lachmann A, et al: **Enrichr: a comprehensive gene set enrichment analysis web server 2016 update.** *Nucleic Acids Res* 2016, **44:**W90-97.
20. Qiu X, Hill A, Packer J, Lin D, Ma YA, Trapnell C: **Single-cell mRNA quantification and differential analysis with Census.** *Nat Methods* 2017, **14:**309-315.
21. Efremova M, Vento-Tormo M, Teichmann SA, Vento-Tormo R: **CellPhoneDB: inferring cell-cell communication from combined expression of multi-subunit ligand-receptor complexes.** *Nat Protoc* 2020, **15:**1484-1506.
22. Hwang WL, Jagadeesh KA, Guo JA, Hoffman HI, Yadollahpour P, Reeves JW, Mohan R, Drokhlyansky E, Van Wittenberghe N, Ashenberg O, et al: **Single-nucleus and spatial transcriptome profiling of pancreatic cancer identifies multicellular dynamics associated with neoadjuvant treatment.** *Nat Genet* 2022, **54:**1178-1191.
23. Weinstein JN, Collisson EA, Mills GB, Shaw KRM, Ozenberger BA, Ellrott K, Shmulevich I, Sander C, Stuart JM, Network CGAR: **The Cancer Genome Atlas Pan-Cancer analysis project.** *Nature Genetics* 2013, **45:**1113-1120.
24. Wang X, Park J, Susztak K, Zhang NR, Li M: **Bulk tissue cell type deconvolution with multi-subject single-cell expression reference.** *Nat Commun* 2019, **10:**380.
25. Aibar S, Gonzalez-Blas CB, Moerman T, Huynh-Thu VA, Imrichova H, Hulselmans G, Rambow F, Marine JC, Geurts P, Aerts J, et al: **SCENIC: single-cell regulatory network inference and clustering.** *Nat Methods* 2017, **14:**1083-1086.
